# Promotion of mature angiogenesis in ischemic stroke by Taohong Siwu decoction through glycolysis activation

**DOI:** 10.3389/fphar.2024.1395167

**Published:** 2024-06-18

**Authors:** Linfeng Tang, Zhuqing Liu, Zhaojie Ji, Xueting Zhang, Mengdie Zhao, Daiyin Peng, Lan Han

**Affiliations:** ^1^ Department of Pharmacy, Anhui University of Chinese Medicine, Hefei, China; ^2^ Anhui Province Key Laboratory of Chinese Medicinal Formula, Hefei, China; ^3^ MOE-Anhui Joint Collaborative Innovation Center for Quality Improvement of Anhui Genuine Chinese Medicinal Materials, Hefei, China

**Keywords:** ischemic stroke, cerebral ischemia-reperfusion injury, mature angiogenesis, glycolysis, Taohong Siwu decoction

## Abstract

**Backgrounds:** Mature angiogenesis plays a critical role in improving cerebral ischemia-reperfusion injury (CIRI). Glycolysis serves as the primary energy source for brain microvascular endothelial cells (BMECs), whereas other vascular cells rely on aerobic respiration. Therefore, intercellular variations in energy metabolism could influence mature angiogenesis. Taohong Siwu Decoction (THSWD) has demonstrated efficacy in treating ischemic stroke (IS), yet its potential to promote mature angiogenesis through glycolysis activation remains unclear.

**Methods:** In this study, we established a middle cerebral artery occlusion/reperfusion (MCAO/R) model *in vivo* and an oxygen-glucose deprivation/reoxygenation (OGD/R) model *in vitro*. We assessed neuroprotective effects using neurobehavioral scoring, 2,3,5-triphenyltetrazolium chloride (TTC) staining, Hematoxylin-eosin (HE) staining, and Nissl staining in MCAO/R rats. Additionally, we evaluated mature angiogenesis and glycolysis levels through immunofluorescence, immunohistochemistry, and glycolysis assays. Finally, we investigated THSWD’s mechanism in linking glycolysis to mature angiogenesis in OGD/R-induced BMECs.

**Results:** *In vivo* experiments demonstrated that THSWD effectively mitigated cerebral damage and restored neurological function in MCAO/R rats. THSWD significantly enhanced CD31, Ang1, PDGFB, and PDGFR-β expression levels, likely associated with improved glucose, pyruvate, and ATP levels, along with reduced lactate and lactate/pyruvate ratios. *In vitro* findings suggested that THSWD may boost the expression of mature angiogenesis factors (VEGFA, Ang1, and PDGFB) by activating glycolysis, increasing glucose uptake and augmenting lactate, pyruvate, and ATP content, thus accelerating mature angiogenesis.

**Conclusion:** THSWD could alleviate CIRI by activating the glycolysis pathway to promote mature angiogenesis. Targeting the glycolysis-mediated mature angiogenesis alongside THSWD therapy holds promise for IS treatment.

## Graphical Abstract

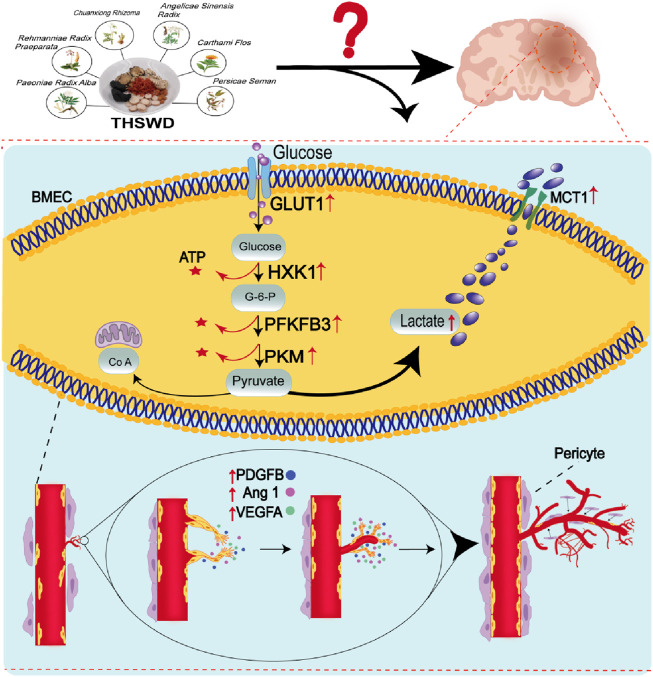

## Introduction

Ischemic stroke (IS) is a prominent cause of mortality and permanent disability worldwide, posing substantial economic and quality of life challenges (GBD, 2019 Stroke Collaborators, 2021; Behera et al., 2020). Current clinical interventions for IS patients are largely limited to interventional therapy or thrombolytic therapy with recombinant tissue plasminogen activator, constrained by a narrow therapeutic window (Behera et al., 2020). Following a stroke, blood flow can reach the ischemic area through collateral or neovascularization, which may compensate for cerebral tissue (Liu D. et al., 2016). The restoration of collateral circulation in the ischemic penumbra is a pivotal factor in stroke prognosis (Chng et al., 2008), underscoring the potential of promoting collateral formation and angiogenesis in IS treatment.

However, compensatory neovascularization in stroke patients is often limited, with the risk of vessel no-reflow or exacerbated cerebral edema (Li et al., 2007). Consequently, there is considerable interest in promoting angiogenesis in ischemic regions through exogenous gene, protein, or drug supplementation (Kumar et al., 2008). Notably, localized brain injections of VEGFA for therapeutic angiogenesis have shown limitations, including high permeability and low perfusion in neovascularization (Zhang et al., 2000; Zhang et al., 2017). Consequently, while focusing on the number of neovascularization, we should pay more attention to the maturity and functionality of the neovascularization. Thus, the focus has shifted towards enhancing the maturity and functionality of neovascularization.

Mature angiogenesis is a highly dynamic process involving endothelial cell (EC) differentiation, proliferation, migration, pericyte recruitment, and basement membrane remodeling, culminating in the formation of perfused vessels in ischemic areas (Chang et al., 2017; Gete et al., 2021). EC hyperglycolysis, akin to the Warburg effect in tumors, plays a critical role in this process (Cruys et al., 2016). Studies have shown that manipulating glycolysis can modulate angiogenesis; for instance, PFKFB3 gene knockdown inhibits EC glycolysis and angiogenesis (Schoors et al., 2014), while increased hexokinase expression boosts glycolysis and promotes angiogenesis (Yu et al., 2017).

Additionally, ECs recruit pericytes to stabilize vascular structures (Armulik et al., 2011), with other vascular cells primarily relying on aerobic respiration (Cunnane et al., 2020). Ischemia and hypoxia stimulate increased glycolysis, promoting EC proliferation and differentiation but potentially impairing other vascular cell functions. Thus, understanding the interplay and molecular mechanisms of ECs glycolysis levels in mature angiogenesis after IS warrants further investigation.

Traditional Chinese medicine (TCM) views IS as a manifestation of blood stasis syndrome, focusing on “stasis-removing and regeneration-promoting” in treatment (Liu et al., 2016; Li et al., 2017). Taohong Siwu Decoction (THSWD), a classic TCM formulation for this purpose, consists of *Prunus persica* (L.) Batsch, *Carthamus tinctorius* L, *Angelica sinensis* (Oliv.) Diels, *Rehmannia glutinosa* (Gaertn.) DC, *Ligusticum chuanxiong* Hort, *Paeonia lactiflora* Pall, as documented in the Golden Mirror of Medicine of the Qing Dynasty (Xia et al., 2021). Our previous research has shown that TSHWD effectively treats cerebral ischemia-reperfusion injury (CIRI) (Li et al., 2015; Chen et al., 2020; Wang et al., 2020), and it promotes angiogenesis by increasing VEGFA, CD34, and BrdU/vWF levels in the ischemic penumbra (Chen et al., 2020). Therefore, we aim to investigate whether THSWD alleviates CIRI by promoting mature angiogenesis and whether this involves the activation of the ECs glycolysis pathway.

To address these questions, we utilized middle cerebral artery occlusion/reperfusion (MCAO/R) *in vivo* and oxygen-glucose deprivation/reoxygenation (OGD/R) *in vitro* models. We examined the role of THSWD in mature angiogenesis after IS and explored the underlying molecular mechanisms involving glycolysis pathways.

## Materials and methods

### Herbs and reagents

The details of the six herbs comprising THSWD are presented in Table 1. These herbs were sourced from Anhui Bozhou Xiehecheng Pharmaceutical Co., Ltd. Butylphthalide (NBP, 1182110134) was acquired from Shiyao Group Enbip Pharmaceutical Co. The 2% 2,3,5-triphenyltetrazolium chloride (TTC, BL1215A) staining solution was obtained from Beijing G-CLONE Biotechnology Co, and the Hematoxylin-eosin (HE) stain (G1003) kit was purchased from Wuhan Servicebio Biotechnology Co. Additionally, the Nissl staining kit (10262109) was procured from Hefei eBioGo Biotechnology Co. For biochemical analyses, glucose (012522220429), lactate (20020421), pyruvate (20020525), and ATP (20220919) kits were acquired from Nanjing Jianjian Biotechnology Co.

**TABLE 1:** The basic information of THSWD.

Herbal name	Plant scientific name	Lot number	Weight ratio	Representative components
*Paeoniae Radix Alba*	*Paeonia lactiflora* Pall	22073002	3	paeoniflorin
*Rehmanniae Radix Praeparata*	*Rehmannia glutinosa* (Gaertn.) DC.	23010311	4	verbascoside
*Angelicae Sinensis Radix*	*Angelica sinensis* (Oliv.) Diels	23070403	3	ferulic acid
*Chuanxiong Rhizoma*	*CLigusticum chuanxiong* Hort	22070701	2	ligustilide
*Persicae Seman*	*Prunus persica* (L.) Batsch	23060504	3	amygdalin
*Carthami Flos*	*Carthamus tinctorius* L	23060504	2	hydroxysafflor yellow A

Primary antibodies, including CD31 (25u3337), PDGFR-β (01052356), PDGFB (57n6785), and Ang1 (00081292), were purchased from Wuhan Sanying Biotechnology Co. The 2-Deoxy-D-glucose (2DG) (156249) was sourced from Shanghai MCE Biotechnology Ltd., and the Matrigel matrix (356234) was obtained from Corning Incorporated, United States. Enzyme-linked immunosorbent assay (ELISA) kits for Ang1, PDGFB, and VEGFA (202210) were obtained from Shanghai Jianglai Biotechnology Ltd. Furthermore, the primary antibodies, GLUT1 (B1157), HXKI (B1218), PKM (I3022), MCT1 (D0522), and PFKFB3 (D1022) were procured from Santa Cruz, United States.

### THSWD preparation and quality control

The specified amounts of herbs were weighed according to the proportions outlined in Table 1. They were initially decocted in 10 volumes of water for 2 h, followed by filtration and storage. Subsequently, a second decoction was carried out using 8 volumes of water for 1.5 h, and the resulting filtrate was combined with the first batch and concentrated to a density of 1.8 g/mL (Pan et al., 2022).

Furthermore, quality control of THSWD was conducted using ultra-performance chromatography-mass spectrometry (UPLC MS/MS), adhering to established experimental protocols (Chen et al., 2020).

### MCAO/R modeling

All Sprague-Dawley (SD) rats (SCXK 2019-0003) used in this study were procured from the Laboratory Animal Center of Anhui University of TCM (Ethics Committee No. AHUCM-rats-2021041) and weighed approximately 250 g ± 20 g. The MCAO/R model was induced using the thread-embolism technique (Wu et al., 2020). Briefly, rats were anesthetized with intraperitoneal pentobarbital injection (30 mg/kg), followed by isolation of the common carotid artery, external carotid artery, and internal carotid artery. A small incision was made in the common carotid artery, and a thread embolus (0.40 ± 0.02 mm) was inserted into the internal carotid artery approximately 18–22 mm. The sham group underwent the same procedure without artery ligation or thread embolus insertion. After 2 h of ischemia, the thread embolus was removed to restore perfusion for 24 h, and the efficacy of the model was evaluated using the Zea Longa score. The rats were then randomly divided into sham, model, and THSWD groups (4.5 g/kg, 9 g/kg, 18 g/kg), as well as a group receiving NBP (25 mg/kg). Subsequently, the corresponding indexes were assessed following 7 days of continuous drug or saline gavage for each group.

### Neurological function score

After completing the treatment protocol, the rats underwent neurological assessments using the Zea Longa score (Li et al., 2023) and the modified neurologic severity score (mNSS) (Chen et al., 2001). The Zea Longa score was assigned as follows: 0 for normal walking, 1 for inability to extend the left forelimb, 2 for tilt walking, 3 for counterclockwise circling, and 4 for inability to walk or unconsciousness. The mNSS, as previously described, assigns a higher score (ranging from 0 for normal function to 18 for maximal deficit) to indicate more severe neurological impairment. All assessments of animal neurological function were conducted by trained researchers who were unaware of grouping information.

### Cerebral infarction volume

The rat brain tissue was washed with pre-cooled PBS and sliced into 2 mm sections along the sagittal plane. These brain slices were then incubated in a 2% TTC staining solution for 30 min at 37°C, protected from light. Subsequently, the slices were immersed in 4% paraformaldehyde for 24 h. The cerebral infarct volume was quantified using ImageJ software.

### HE staining and Nissl staining

Fresh brain tissues were fixed in 4% paraformaldehyde for 24 h and then embedded in paraffin to prepare 4 mm coronal sections. HE staining and Nissl staining were conducted according to standard protocols. Brain tissue damage was assessed using a light microscope. Additionally, Nissl bodies were quantitatively analyzed using ImageJ software.

### Biochemical kits and ELISA

Glucose, pyruvate, and lactate levels were determined in rat serum and cell culture media using biochemical kits. Additionally, the VEGFA, Ang1, and PDGFB levels in cell culture media were measured using ELISA kits. Protein concentrations in cerebral cortical tissue from the ischemic area and cellular extracts were quantified separately using a BCA kit. Furthermore, the glucose, pyruvate, lactate, and ATP levels in the cerebral cortex and cellular ATP content were assessed using biochemical assay kits. All procedures were conducted in accordance with the manufacturers’ instructions.

### Immunofluorescence

The rat brains were swiftly extracted to prepare frozen sections. The sections were incubated with anti-CD31 (1:500) antibodies at 4°C overnight and restained with DAPI for 5 min following incubation with secondary antibodies (1:1000). All images were acquired using a fluorescence microscope (Nikon/Eclipse, Tokyo, Japan), and the fluorescence intensity was assessed using ImageJ.

### Immunohistochemistry

The cerebral specimens underwent fixation, paraffin embedding, section preparation, programmed dewaxing, dehydration, antigen repair, and sealing procedures. Subsequently, the sections were incubated overnight at 4°C with primary antibodies against Ang1 (1:100), PDGFB (1:200), and PDGFR-β (1:200). The following day, the specimens were washed with PBS and then incubated with secondary antibodies (1:1000) for 30 min at room temperature. They were subsequently stained using DAB and hematoxylin before being sealed for photograph collection. In parallel, mature angiogenesis protein expression was analyzed using ImageJ software.

### THSWD drug-containing serum preparation

SD rats were administered THSWD (1.8 g/mL) via oral gavage twice daily for three consecutive days. Rats in the normal group received saline supplementation instead. Following the final THSWD administration, rats were anesthetized, and blood samples were immediately collected from the abdominal aorta. The samples were allowed to stand for 2 hours, followed by centrifugation at 3000 rpm for 20 min at 4°C. The supernatant was then heated in a 56°C water bath for 30 min for inactivation, filtered through a 0.22 μm microporous filter membrane for sterilization, and stored at −80°C until analysis. The analysis of blood components was performed according to established protocols detailed in our previous research (Duan et al., 2020).

### OGD/R

Brain microvascular endothelial cells (BMECs) were obtained from the BeNa Culture Collection (337717), while rat brain microvascular pericytes were purchased from iCell Bioscience Inc. (2022092701). Both cell types were cultured in high glucose DMEM medium with 10% FBS in a standard cell culture incubator (5% CO_2_, 95% O_2_). To simulate CIRI, BMECs were exposed to glucose-free DMEM medium and placed in a hypoxic incubator (1% O_2_, 5% CO_2_ with 94% N_2_) for 4 hours (Shi et al., 2023). Subsequently, the medium was replaced with glucose-containing DMEM, and the cells were transferred to a standard cell culture incubator for 24 h of reoxygenation.

### Drug concentration screening and grouping

A total of 100 μL of BMECs (1 × 10^5^ cells/mL) were seeded into each well of a 96-well plate and randomly assigned to either a normal group or an OGD/R group. Following 4 hours of OGD, each group received different concentrations of THSWD drug-containing serum and 2DG. After 24 h of reoxygenation, 10 μL CCK8 solution was added to each well and incubated for 1 h, with the optical density (OD) value measured at 450 nm.

To further assess the impact of THSWD on glycolysis levels and angiogenesis in OGD/R-induced BMECs, cells were categorized into the following groups: normal group (10% blank serum), OGD/R group (10% blank serum), THSWD group (OGD/R+10% THSWD drug-containing serum), 2DG group (OGD/R+10% blank serum+5 mM 2DG), and 2DG + THSWD group (OGD/R+10% THSWD drug-containing serum+5 mM 2DG).

### Mature angiogenesis

#### CCK8

The proliferation of BMECs in the normal and drug-processed groups was assessed using the same procedure as the previous description of the CCK8 approach.

#### Scratch test

BMECs were seeded in 6-well plates and grown to the shape of a “paving stone road.” After an OGD treatment of 4 h, scratches were made using a sterile toothpick. After cleaning with PBS, the 0-h scratch locations were marked and photographed for recording. After 24 h of reoxygenation treatment, pictures of recordings were retaken, and the cell migration rate of all groups was examined using ImageJ.

#### Sprouting test

The sphere sprouting assay of BMECs was performed with slight modifications (De Bock et al., 2013). We spread the cell culture dish using 0.5% sterile agarose solution. After the solution was cooled, 3 mL of BMECs (1 × 10^5^ cells/mL) were seeded to form cell spheroids. We combined the rat tail tendon collagen type I and M199 medium in a 10:1 ratio to produce a light yellow solution. Next, we immediately transferred the cell spheroid suspension to the light yellow solution and allowed it to stand for 2 hours in the cell culture incubator. Finally, we conducted OGD/R modeling and observed the sphere sprouting after 24 h. Additionally, the length of the sphere sprouting and the number of sprouts were assessed using ImageJ.

#### Tube formation assay

We inoculated 50 μL of precooled Matrigel matrix into 96-well plates (Liu et al., 2023). After modeling and drug intervention, we digested the BMECs, along with 100 μL (1 × 10^5^ cells/mL) of suspension per well, and transferred it to the Matrigel matrix. After culturing for 3 h, photographs were taken to record the tube formation in each group. ImageJ analysis was performed to assess the tube number and branching.

#### Transwell assay

The day preceding the assay, 1 mL of pericytes (1 × 10^5^ cells/mL) and BMECs (1 × 10^5^ cells/mL) were seeded in the upper and lower chambers of the Transwell, respectively, and allowed to incubate overnight. The following day, both upper and lower chamber cells were recycled with glucose-free medium and placed in a low-oxygen incubator for 4 hours. We changed the upper pericytes to a culture medium supplemented with 1% FBS, and the lower chamber BMECs were changed to a culture medium containing 10% FBS. They were incubated in a normal cell culture incubator for 24 h and fixed using 4% paraformaldehyde for 30 min before staining with 0.5% crystal violet staining solution. After photographs were taken, they were analyzed using ImageJ to assess the number of pericyte migrations.

#### Western Blotting (WB)

The BMECs were lysed in a pre-cooled RIPA solution containing PMSF for 20 min to obtain total proteins. We measured the protein concentration using a BCA kit and included a consistent protein content in each group. Using 10% or 12% SDS-PAGE electrophoresis for 2 hours, the proteins were transferred to an NC membrane for 1 hour. After incubating with 5% skimmed milk powder for 2 hours and primary antibody overnight at 4°C, the membranes were washed three times with TBST, and incubated with HRP-labeled secondary antibody for 2 hours for chemiluminescence reaction.

#### Statistical analysis

All experimental findings were presented using Mean ± SD. Comparisons between two groups were made using a two-tailed Student's t-test, while comparisons between three or more groups were performed using a one-way analysis of variance. SPSS 25.0 software was adopted for statistical analysis. *p* < 0.05 was considered statistically significant.

## Results

### UPLC MS/MS profile of THSWD

**FIGURE 1:**
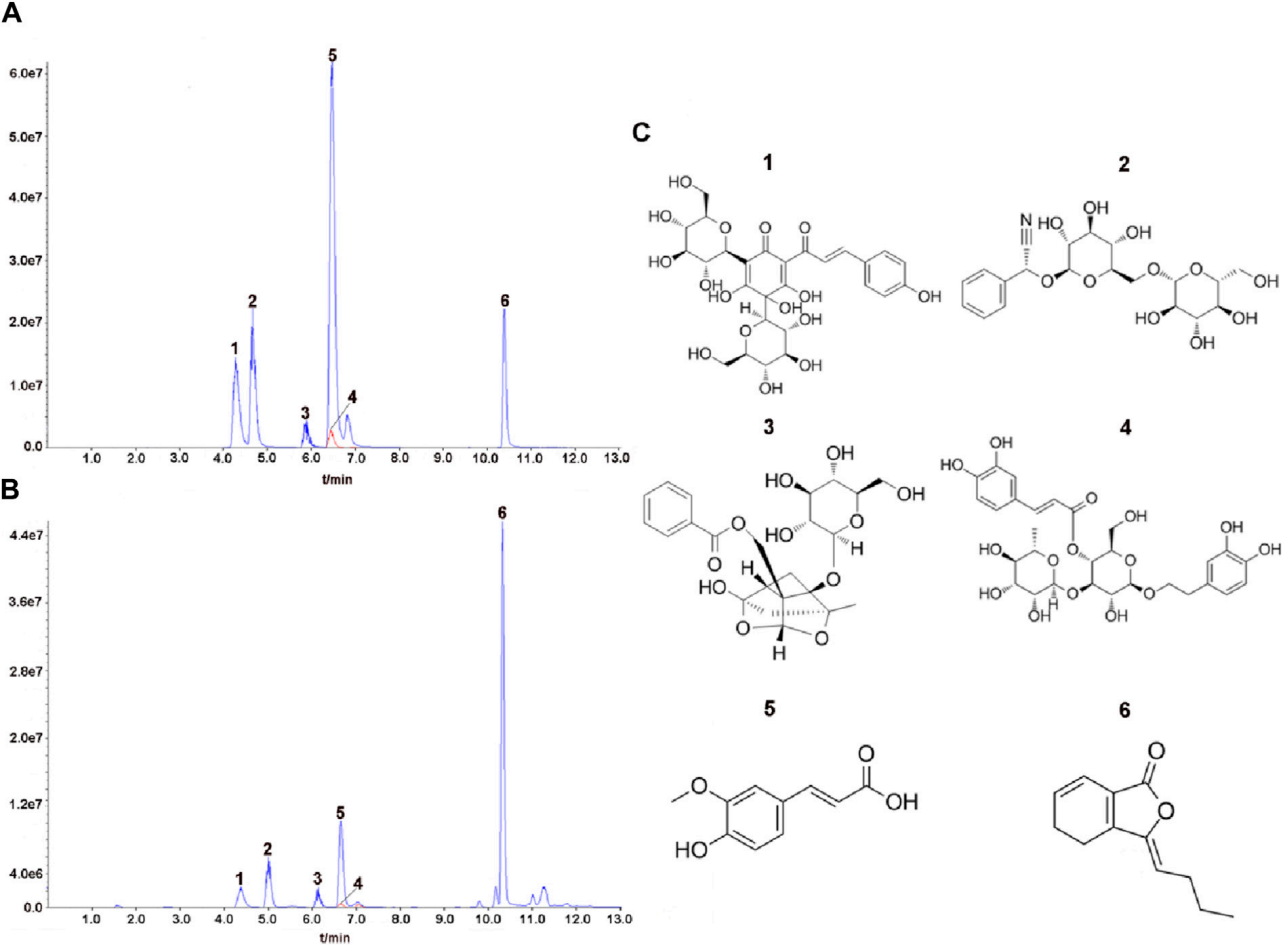
Analysis of representative components of THSWD using UPLC MS/MS. The chromatograms of six representative components of the mixed standard compounds **(A)** and THSWD **(B)**. **(C)** The chemical structures of six representative components. 1: hydroxysafflor yellow A; 2: amygdalin; 3: paeoniflorin; 4: verbascoside; 5:ferulic acid; 6:ligustilide.

A total of six THSWD-indicative components were characterized using the UPLC MS/MS approach (Figures 1A–C). We calculated the contents of the THSWD (1 mg/mL) components according to the standard curve method: hydroxysafflor yellow A (2.572 μg/mL), amygdalin (6.793 μg/mL), paeoniflorin (25.374 μg/mL), verbascoside (0.165 μg/mL), ferulic acid (0.989 μg/mL), and ligustilide (8.023 μg/mL). The mass spectrometry data was shown in Supplementary Material 1.

### THSWD ameliorated cerebral ischemia and neurological function in MCAO/R rats

**FIGURE 2:**
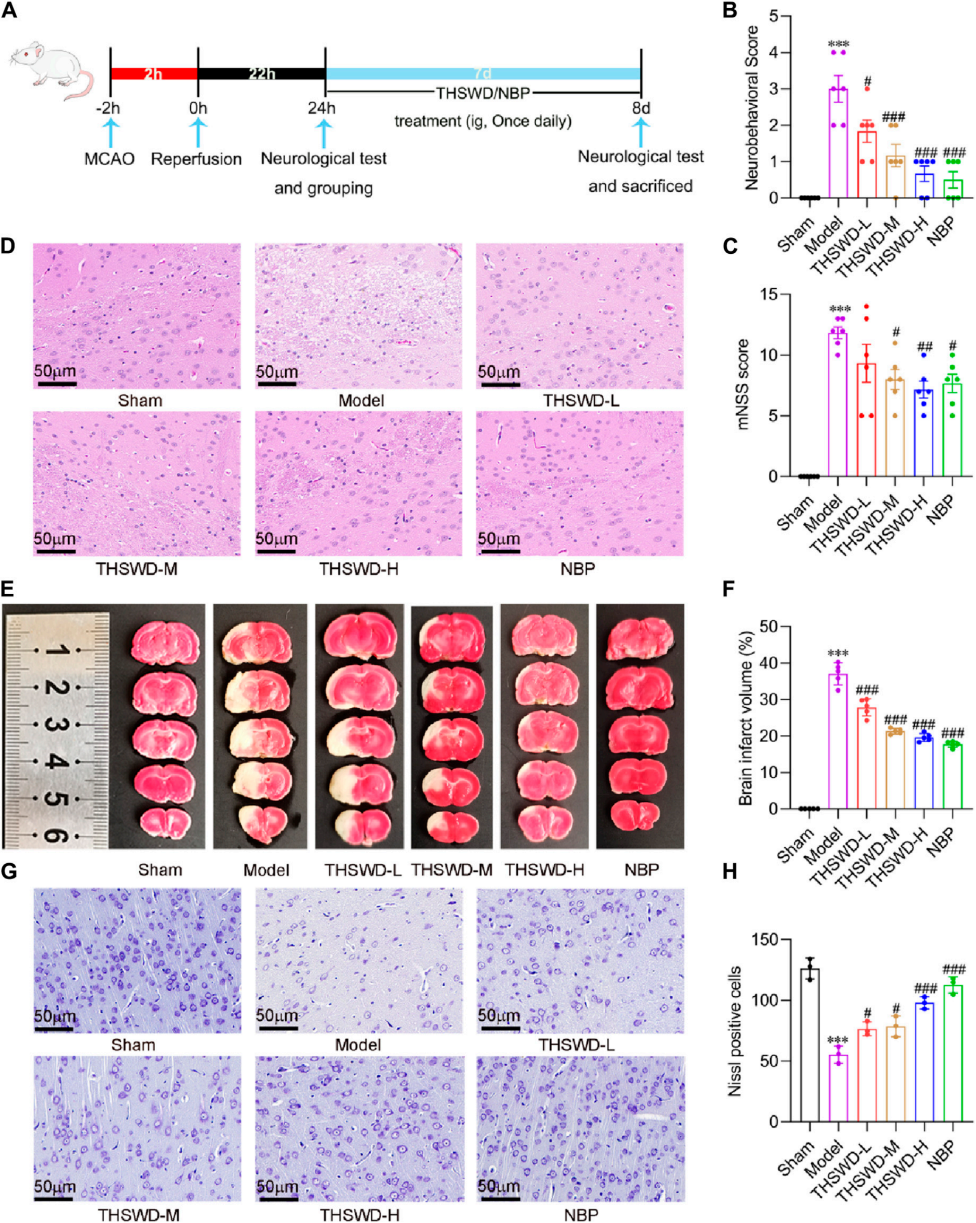
The effects of THSWD on neurological function, cerebral infarct volume, histopathology, and neuronal injury in MCAO/R rats. **(A)** The flow diagram of the experiment. **(B)** Neurobehavioral score (*n* = 6). **(C)** mNSS score (*n* = 6). **(D)** Representative images of HE staining of the cerebral cortex on the Ischemia side (*n* = 3). **(E)** Representative images of TTC staining. **(F)** Quantification of cerebral infarct volume (*n* = 5). **(G)** Representative images and quantitative analysis of Nissl bodies **(H)** in the cerebral cortex of the ischemic side. The scale bar represents 50 μm. Data were expressed as mean ± SD. ^***^ *p* < 0.001 vs. the sham group; ^#^ *p* < 0.05, ^##^ *p* < 0.01, ^###^ *p* < 0.001 vs. the model group.

After developing the MCAO/R model, we administered treatment by supplementing varying doses of THSWD and NBP (Figure 2A). Compared to the Sham group, MCAO/R modeling significantly increased Zea Longa and mNSS scores. After the intervention, the Zea Longa and mNSS scores in the THSWD group were significantly lower than those in the MCAO/R group (Figures 2B,C). HE results suggested that the brain tissue in the ischemic area of rats in the model group possessed severe vacuolation and necrosis. Conversely, THSWD could effectively alleviate pathologic damage (Figure 2D). The milky white area denotes the site of cerebral infarction. The TTC results indicated that the rats in the model group had prominent areas of cerebral infarction (Figure 2E). Differing doses of THSWD significantly reduced cerebral infarct volume (Figures 2E,F). We used Nissl staining to evaluate the neuronal damage, finding that THSWD significantly increased the number of Nissl bodies in the ischemic cortical region of MCAO/R rats and improved cerebral neurological impairment (Figures 2G,H). In conclusion, THSWD exhibited a favorable neuroprotective effect on MCAO/R rats.

### THSWD accelerated mature angiogenesis in MCAO/R rats

**FIGURE 3:**
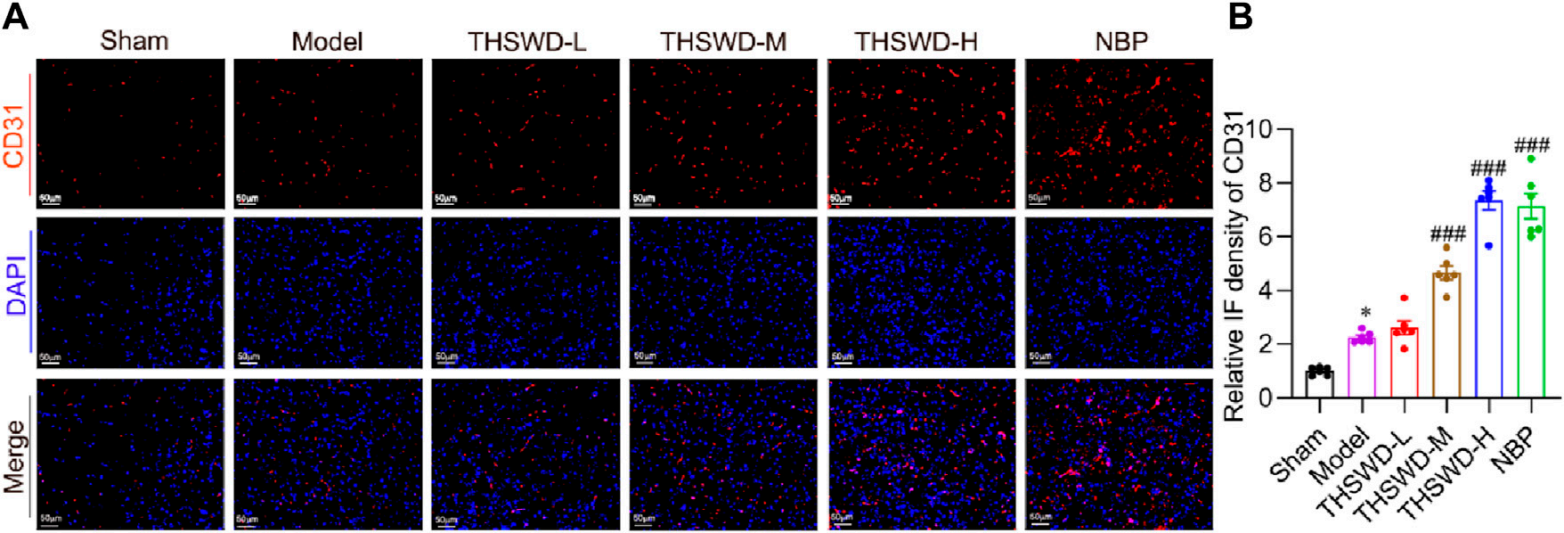
THSWD accelerated angiogenesis in MCAO/R rats. **(A)** Representative images of CD31 detected by Immunofluorescence staining and **(B)** quantitative analysis (*n* = 6). The scale bar represents 50 μm. Data were expressed as mean ± SD. ^*^ *p* < 0.05 vs. the sham group; ^*###*^ *p* < 0.001 vs. the model group.

As previously identified, promoting mature angiogenesis may be critical for improving CIRI. To confirm the microvessel density, the expression of CD31 in ischemic areas was detected using immunofluorescence staining (Hui et al., 2022). The immunofluorescence findings indicated that the expression of CD31 was slightly increased in the MCAO/R group, but it was significantly elevated after the administration of THSWD (Figures 3A,B).

**FIGURE 4:**
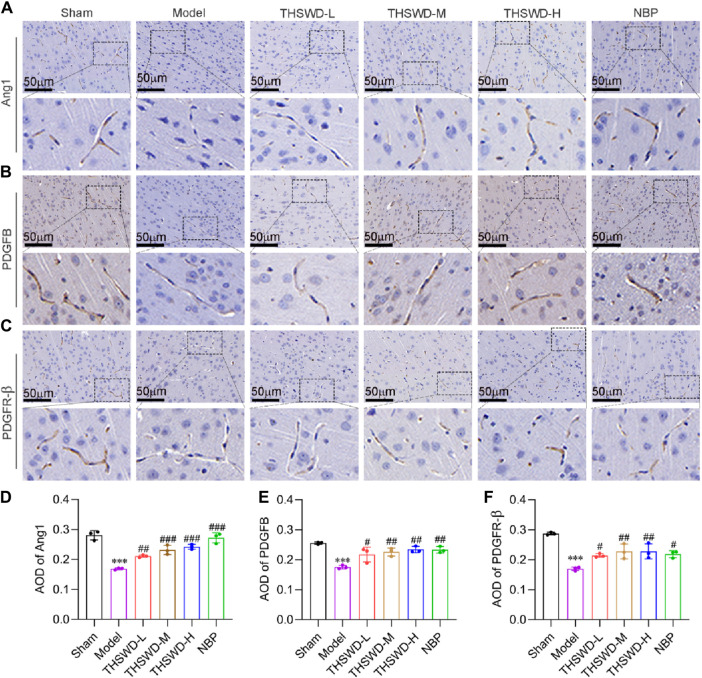
THSWD promotes the expression of mature angiogenesis factors in MCAO/R rats. Representative images of **(A)** Ang1, **(B)** PDGFB, and **(C)** PDGFR-β in the cortical vessels on the ischemic side of the cerebral in MCAO/R rats and **(D–F)** corresponding quantitative analysis. The scale bar represents 50 μm. Data were expressed as mean ± SD (*n* = 3). ^***^ *p* < 0.01 vs. the sham group; ^#^ *p* < 0.05, ^##^ *p* < 0.01, ^###^ *p* < 0.001vs. the model group.

Pericyte recruitment can increase vascular structural stability and accelerate neovascularization maturation (Armulik et al., 2011). Ang1 is primarily expressed in growing vascular ECs and pericytes, attracting pericyte recruitment upon binding to Tie2 receptors on ECs (Lee et al., 2009; Koh, 2013). While PDGFB is mainly secreted by ECs, interacting with PDGFR-β expressed by pericytes, which promotes pericyte aggregation, proliferation and migration (Hellström et al., 2001). Immunohistochemical results demonstrated that the expression levels of Ang1, PDGFB, and PDGFR-β were significantly reduced in the ischemic region of MCAO/R rats. Conversely, treatment with THSWD effectively elevated the expression of the indicators (Figures 4A–F), suggesting that THSWD may improve CIRI by promoting mature angiogenesis.

### THSWD activated the glycolysis pathway in MCAO/R rats

**FIGURE 5:**
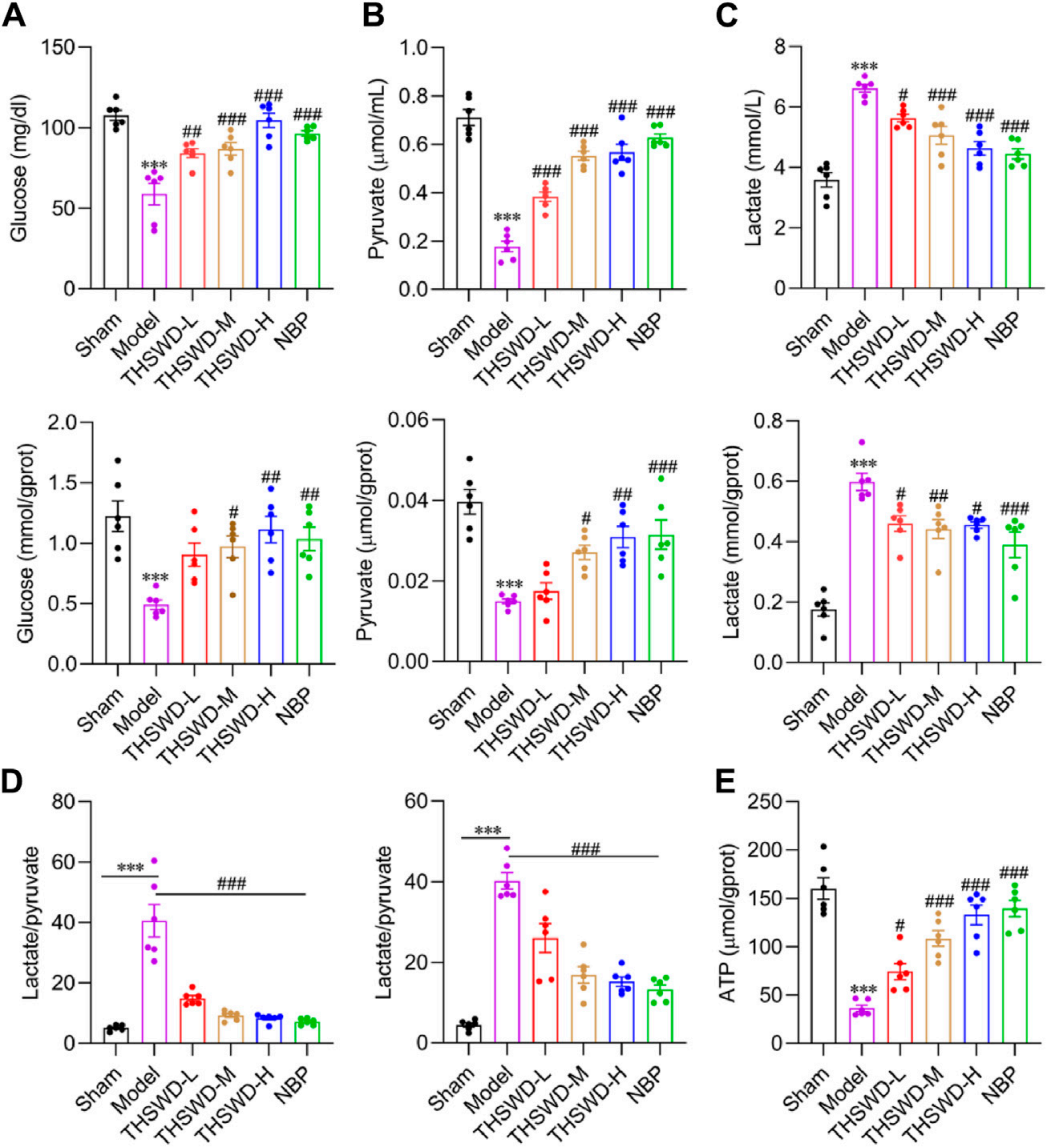
Effect of THSWD on glycolysis levels in MCAO/R rats. The glycolysis kit assays the expression of glucose **(A)**, pyruvate **(B)**, lactate **(C)**, and lactate/pyruvate **(D)** in the serum and the ischemic side cortex of MCAO/R rats. **(E)** Cortical ATP content in the ischemic side. Data were expressed as mean ± SD (*n* = 6). ^***^ *p* < 0.01 vs. the sham group; ^#^ *p* < 0.05, ^##^ *p* < 0.01, ^###^ *p* < 0.001 vs. the model group.

Diversity in energy metabolism across different cells may be a significant cause of defective vascular function. The products of aerobic and anaerobic glycolysis of glucose are pyruvate and lactate, respectively (Helms et al., 2007). The lactate/pyruvate levels reflect the degree of ischemia and hypoxia, with higher ratios indicating worsened ischemia and hypoxia (Raudam and Rivis, 1976; Chen et al., 2000). Compared to the sham group, the serum and ischemic cortex’s glucose (Figure 5A) and pyruvate (Figure 5B) levels in model rats were significantly lower. Conversely, the lactate content (Figure 5C) and the lactate/pyruvate ratio (Figure 5D) were significantly higher. Supplementing THSWD reverses the alterations in the contents of the above indexes. The variation in ATP content further confirms our hypothesis (Figure 5E). THSWD may promote the vascular differentiation of BMECs into vessels via activation of glycolysis while restoring the recruitment function of pericytes, facilitating mature angiogenesis. However, the molecular mechanisms of glycolysis and mature angiogenesis and the interventional effects of THSWD remain unclear.

### THSWD enhanced glycolysis level in OGD/R-induced BMECs

**FIGURE 6:**
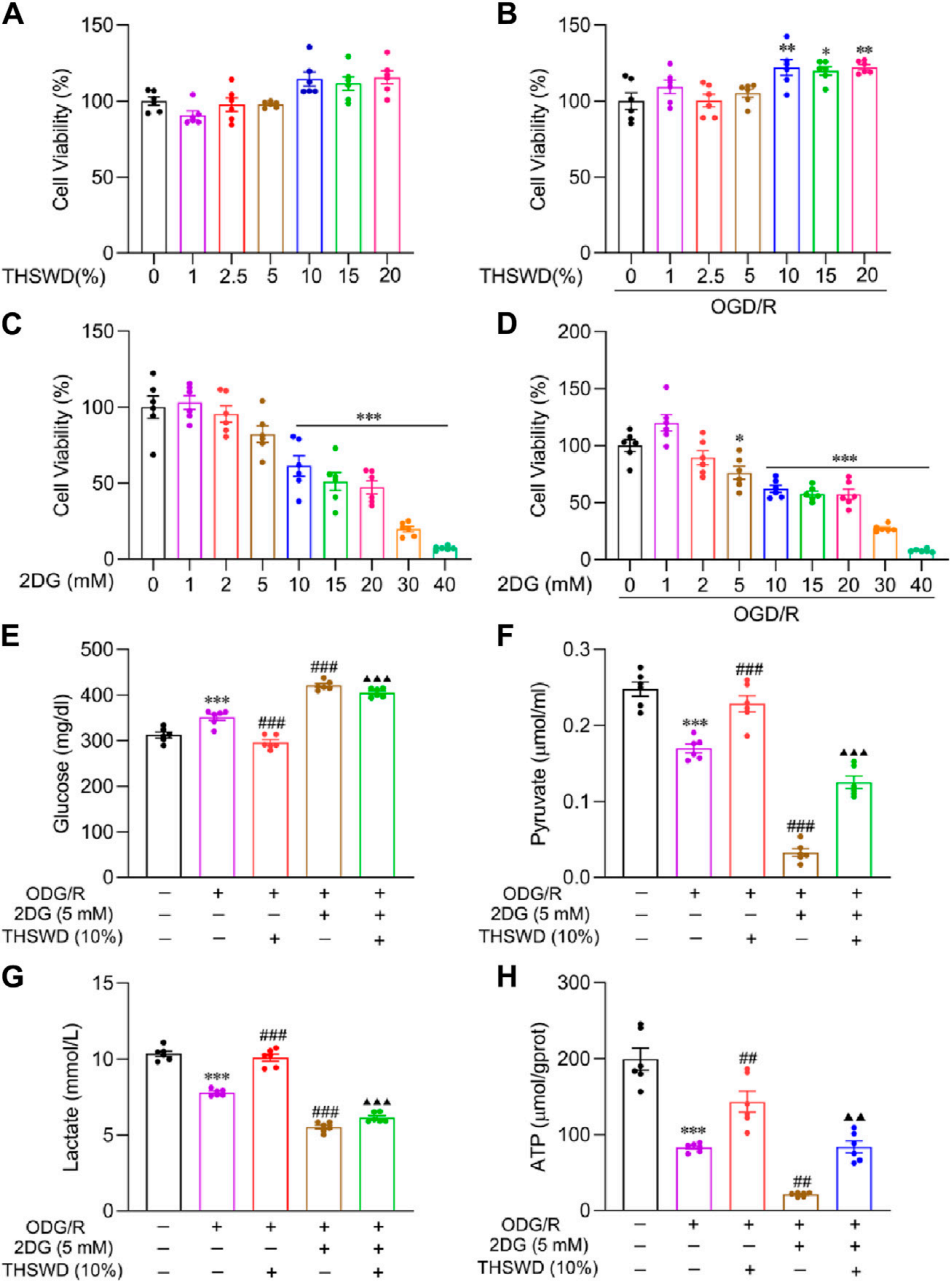
The effect of THSWD on glycolysis levels in OGD/R-induced BMECs. Effects of THSWD and 2DG on survival of BMECs under **(A, C)** normal and **(B, D)** OGD/R. The glycolysis Kit assays the **(E)** glucose, **(F)** pyruvate, **(G)** lactate, and **(H)** intracellular ATP content of the culture medium. Data were expressed as mean ± SD (*n* = 6). ^*^ *p* < 0.05, ^**^ *p* < 0.01, ^***^ *p* < 0.001 vs. the normal group; ^##^ *p* < 0.01, ^###^ *p* < 0.001 vs. the OGD/R group; ^▲▲^ *p* < 0.01, ^▲▲▲^ *p* < 0.001 vs. the THSWD group.

2DG, a glucose analog, inhibits glycolysis by competing for glucose binding to hexokinase (Chen et al., 2022). We integrated the survival rates of BMECs under normal and model groups following pharmacological interventions (Figures 6A–D), determining the concentration of drug administration as THSWD (10%) and 2DG (5 mM).

Glycolysis kit findings demonstrated that glucose uptake (Figure 6E), pyruvate (Figure 6F), lactate release (Figure 6G), and ATP (Figure 6H) content decreased significantly in the OGD/R group relative to the normal group. In addition, THSWD supplementation effectively reversed these indicators. Compared to the OGD/R group, supplementation with 2DG further reduced glycolysis levels, indicating that 2DG has a favorable influence on inhibiting glycolysis. Compared to the THSWD group, the glycolysis level decreased significantly in the 2DG + THSWD group, suggesting that THSWD activated glycolysis.

### THSWD promoted mature angiogenesis in OGD/R-induced BMECs

**FIGURE 7:**
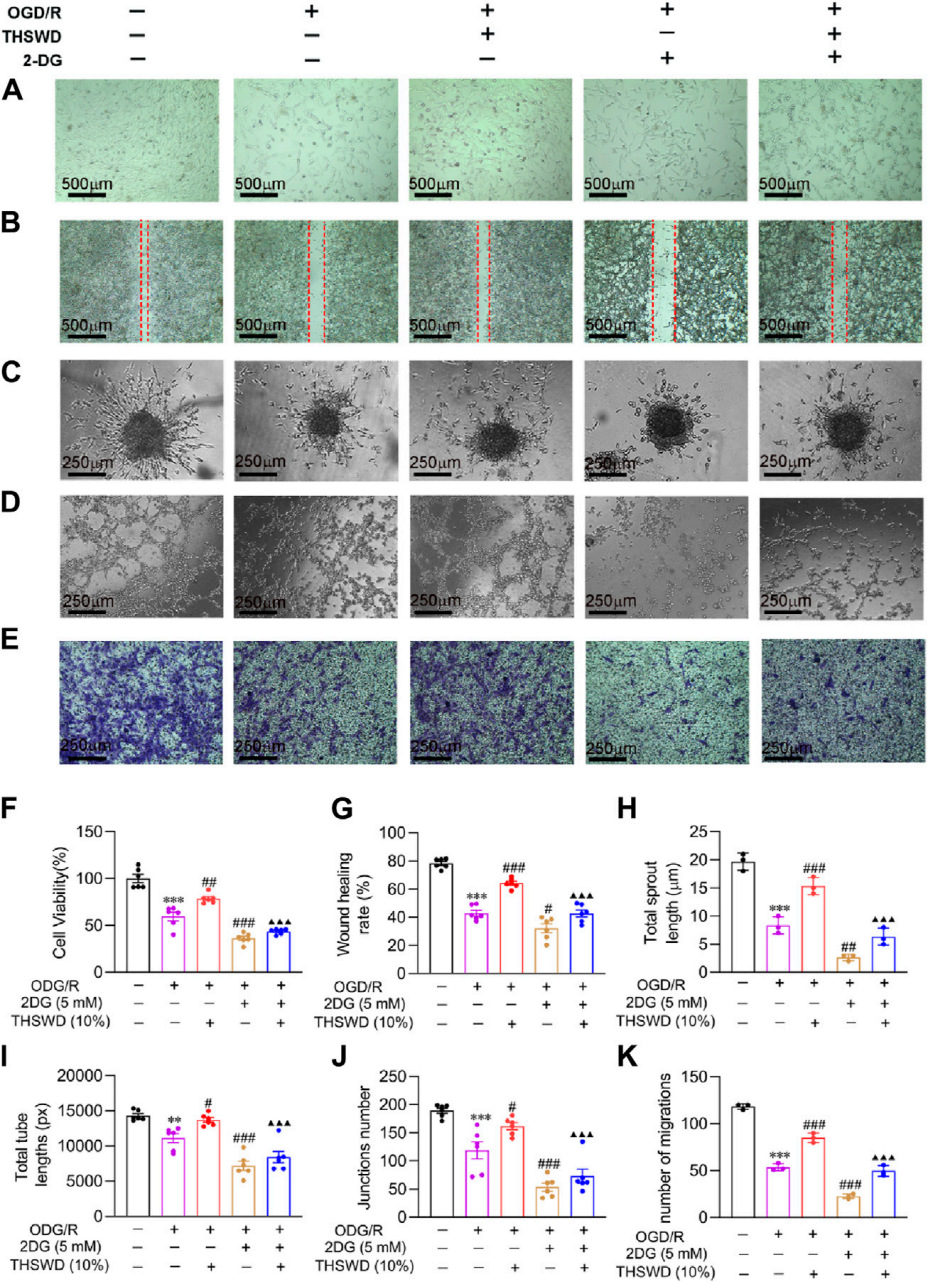
Effect of THSWD on mature angiogenesis of in OGD/R-induced BMECs. **(A)** Morphology (scale bar = 500 μm). **(B)** Wound healing (scale bar = 500 μm). **(C)** Sprouting assay (scale bar = 250 μm). **(D)** Tube formation assay (scale bar = 250 μm). **(E)** Pericyte recruitment (scale bar = 250 μm). **(F)** Quantification of proliferation rate (*n* = 6). **(G)** Wound healing rate (*n* = 6). **(H)** Sprouting length (*n* = 3). **(I,J)** Total tube length and number (*n* = 6). **(K)** Number of pericytes recruited (*n* = 3). Data were expressed as mean ± SD. ^**^ *p* < 0.01, ^***^ *p* < 0.001 vs. the normal group; ^#^ *p* < 0.05, ^##^ *p* < 0.01, ^###^ *p* < 0.001 vs. the OGD/R group; ^▲▲▲^ *p* < 0.001 vs. the THSWD group.

As outlined previously, we employed CCK8, scratch assay, sprouting, tube formation, and transwell assay, to simulate the process of mature angiogenesis *in vivo*. CCK8 and morphological results (Figures 7A, F) demonstrated that the cells in the OGD/R group had a crumpled and floating state, and the number of cells decreased significantly relative to the normal group. Compared to the OGD/R group, the THSWD group significantly improved cell injury and promoted proliferation, while the 2DG group had an aggravated injury state. Compared to the THSWD group, the 2DG + THSWD group substantially attenuated the improvement effect of treatment with THSWD. The scratch assay effectively assesses the migration ability of cells (Sun and Liu, 2022). As illustrated in Figures 7B,G, THSWD may promote the migration of BMECs in the OGD/R model by elevating the glycolysis level. Spheroid sprouting is a central step in angiogenesis, allowing the examination of EC proliferation and division capacity (De Bock et al., 2013). As depicted in Figures 7C,H, THSWD may significantly increase the number of sphere sprouts and sprout length under the OGD/R model by elevating the glycolysis level. The tube formation assay offers an excellent simulation of vascular remodeling *in vivo* (Sun and Liu, 2022). As illustrated in Figures 7D, I, J, THSWD may significantly enhance the tube lengths alongside the number of tube branches in BMECs in the OGD/R model by enhancing glycolysis. Recruitment of pericytes by BMECs is necessary for evaluating vascular maturation (Armulik et al., 2011). As shown in Figures 7E,K, THSWD may elevate the number of pericytes recruited in the OGD/R model by increasing glycolysis levels. Interestingly, it is consistent with the results of pericyte recruitment in animal level. In summary, THSWD promotes mature angiogenesis in BMECs in the OGD/R model by enhancing glycolysis.

### THSWD improved the expression of mature angiogenesis proteins in OGD/R-induced BMECs

**FIGURE 8:**
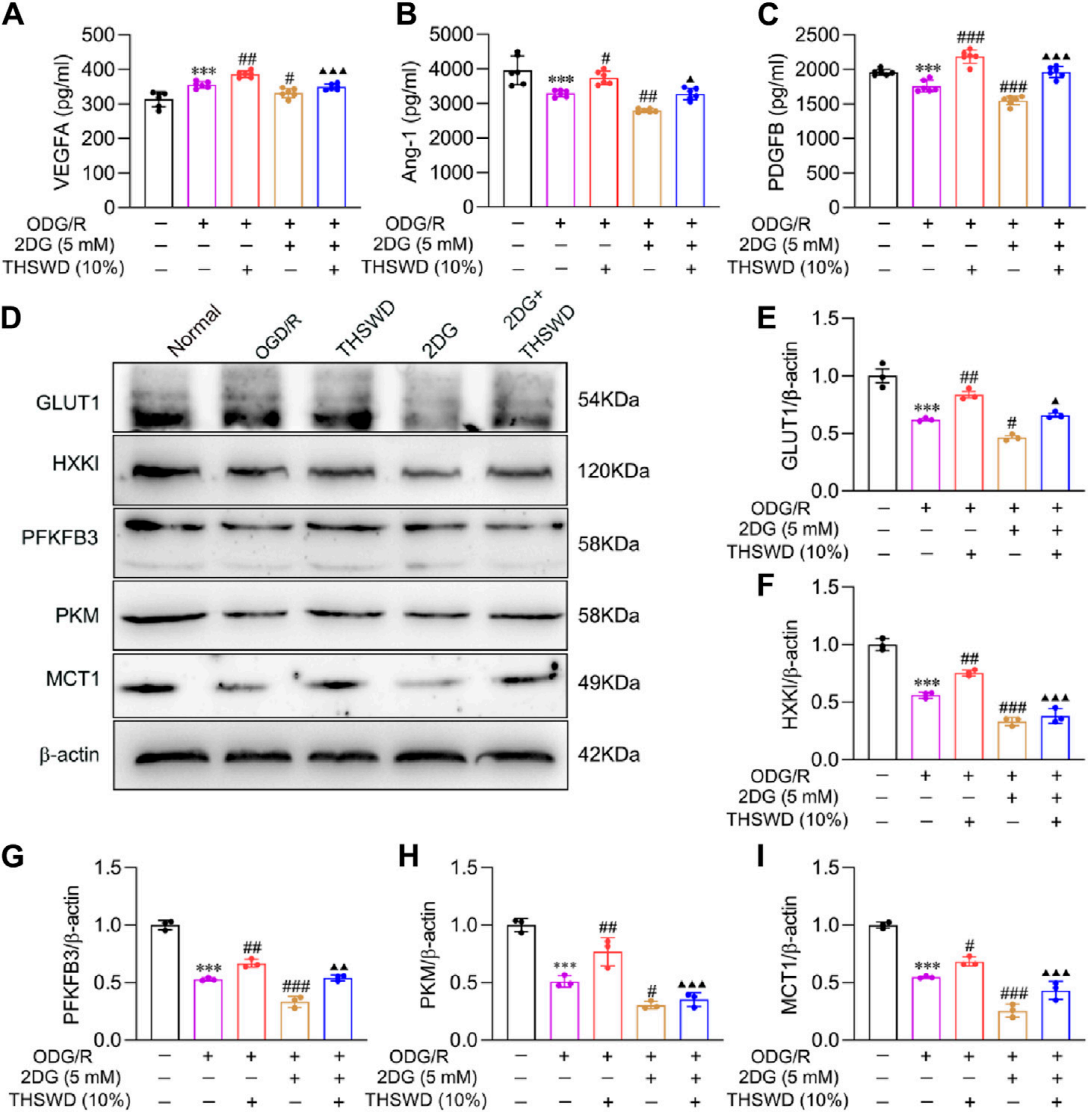
Effect of THSWD on mature angiogenesis factors and glycolysis pathway proteins in OGD/R-induced BMECs. The ELISA detected **(A)** VEGFA, **(B)** Ang1, and **(C)** PDGFB concentrations (*n* = 6). **(D)** The representative protein bands of GLUT1, HXKI, PFKFB3, PKM, and MCT1. **(E–I)** Quantitative statistical results of GLUT1, HXKI, PFKFB3, PKM, and MCT1 (n = 3). Data were expressed as mean ± SD. ^***^ *p* < 0.001 vs. the normal group; ^#^ *p* < 0.05, ^##^ *p* < 0.01, ^###^ *p* < 0.001 vs. the OGD/R group; ^▲^ *p* < 0.05, ^▲▲^ *p* < 0.01, ^▲▲▲^ *p* < 0.001 vs. the THSWD group.

To further illustrate the molecular mechanism of glycolysis and mature angiogenesis, we assayed the expression of mature angiogenesis proteins. VEGFA, an essential regulator of angiogenesis, participates in the process of vascularization (Bikfalvi and Bicknell, 2002). As previously mentioned, the binding of Ang1 to Tie2 and PDGFB to PDGFR-β promotes EC recruitment of pericytes and accelerates vascular remodeling and maturation (Bjarnegård et al., 2004; Zhang et al., 2020). ELISA findings (Figures 8A–C) indicated that the protein content of VEGFA increased significantly in the OGD/R group, while the content of Ang1 and PDGFB decreased significantly relative to the normal group. Compared to the OGD/R group, the 2DG group reduced the expression of mature angiogenesis proteins. Additionally, 2DG also decreases the promotion of THSWD on protein content, indicating that THSWD may enhance the expression of VEGFA, Ang1, PDGFB in BMECs via activation of the glycolysis pathway, promoting mature angiogenesis.

### THSWD regulated glycolysis pathway in BMECs after OGD/R injury

To further elucidate the molecular mechanism of glycolysis activation by THSWD, we examined the expression of glycolysis proteins. The WB results (Figures 8D–I) demonstrated that the glycolysis protein content decreased significantly in the OGD/R group, while it was effectively reversed by THSWD supplementation. Compared to the OGD/R group, the 2DG group could further inhibit glycolysis protein expression, indicating that inhibition of hexokinase could significantly change the expression of upstream and downstream proteins. The above findings indicate that THSWD may hasten glucose uptake into ECs by enhancing GLUT1 expression. Additionally, it increased the activity of the three rate-limiting enzymes, facilitated lactate production, and released huge amounts of ATP. Ultimately, it elevated the expression of MCT1, facilitated lactate efflux to the extracellular compartment, and accelerated the EC glycolysis process.

## Discussion

As angiogenesis can effectively elevate the supply of cerebral blood flow in IS, restore cerebral neurological function, and improve the prognosis of IS patients, some therapeutic strategies promoting angiogenesis have been applied clinically (Chng et al., 2008; Liu X. T. et al., 2016). However, emerging research suggests that neovascularization produced under IS ischemic-hypoxic stimuli, instead of restoring blood flow supply, may exacerbate the development of cerebral edema (Li et al., 2007). This indicates that the function of neovascularization may be more significant than the count of neovessels. Our previous study determined that THSWD could enhance angiogenesis and effectively improve CIRI in MCAO/R rats. Notably, MCAO/R rats promoted angiogenesis yet had little effect on CIRI treatment (Chen et al., 2020). We hypothesized that this might be related to improved neovascularization function by THSWD. BMECs have glycolysis-preferring properties resembling tumor cells (Cruys et al., 2016), while other cell types constituting the neurovascular unit are predominantly aerobically respiratory (Cunnane et al., 2020), attracting a great deal of attention. We hypothesized that the function of mature vessels may be associated with differences in cellular energy metabolism preferences. In this study, we developed the MCAO/R model *in vivo* and the OGD/R model *in vitro*, evaluated the effects of THSWD on mature angiogenesis, and explored its molecular mechanisms.

Mature angiogenesis takes place due to ischemia and hypoxia in the body, and its significant processes involve the initial, progressive, and mature-terminal stages. The process of angiogenesis is modulated by a series of angiogenic regulators (Oyama et al., 1996; Thurston et al., 2000), and under-expression or over-expression of these regulators in the organism may cause defects in vascular function. Following a stroke, the organism increases the content of neovascularization factors like VEGFA, Ang-2, and bFGF during the initial and progressive phases (Oyama et al., 1996; Zhang et al., 2017). However, mature-terminal vascular regulators require further study. Therefore, we hypothesized that the neovascularized no-reflow phenomenon in patients with IS may be associated with defective vascular function due to insufficient expression of mature angiogenic regulators. Our prior study identified that MCAO/R rats with significantly increased levels of angiogenic factors failed to lower the volume of cerebral infarcts, potentially related to the insufficient expression of mature angiogenic factors (Chen et al., 2020). In this study, we identified that the content of mature neovascular factors Ang1 and PDGFB, alongside the number of pericytes recruited (PDGFR-β), were significantly reduced in the model group in both the *in vivo* and *in vitro* assays. The mature angiogenesis factor levels and the number of recruited pericytes were substantially elevated following THSWD supplementation. Therefore, we hypothesized that THSWD may accelerate neovascularization into the mature-terminal phase, increase the number of mature neovessels, and restore cerebral blood flow supply.

Recent studies have demonstrated that the level of glycolysis in BMECs is critical for angiogenesis. Both in the resting state and the activated state, the energy source of BMECs comes primarily from glycolysis rather than oxidative phosphorylation (Perrotta et al., 2020). When the organism is in a hypoxic state, glycolysis can rapidly supply ATP to the migrating and extending filamentous pseudopods (Wang et al., 2019). Conversely, other types of vascular cells, including pericytes, astrocytes, and smooth muscle cells, use oxidative phosphorylation as the primary energy source (Cunnane et al., 2020). Such variability in energy preferences of various types of vascular cells represents a critical factor affecting IS vascular function. The experimental findings of the animal studies indicated that the level of anaerobic glycolysis was remarkably increased in IS model rats, while the ATP content was significantly reduced. After supplementing with THSWD, aerobic glycolysis and the number of recruited pericytes increased, confirming our suspicions. However, the impact of THSWD on glycolysis in BMECs and its molecular mechanisms remain unclear.

EC glycolysis is a complex process encompassing the synergistic action of multiple enzymatic reactions. Studies have uncovered that GLUT1 assists in the passage of glucose through the blood-brain barrier into BMECs and other vascular cells, and its deficiency produces severely impaired energy metabolism (Jurcovicova, 2014). During EC glycolysis, hexokinase, 6-phosphofructokinase-2, and pyruvate kinase are the most prominent regulatory enzymes. Blocking or inhibiting these regulatory enzymes effectively inhibits the EC glycolysis processes (Schoors et al., 2014; Yu et al., 2017). In addition, glycolysis flux mirrors the expression of MCT1 (Végran et al., 2011). The high glycolysis activity of ECs can generate large amounts of accumulated lactate. If not transported promptly, it can inhibit the activity of phosphofructokinase 1, reduce the rate of glycolysis, and even cause glycolysis inactivation in severe cases (Noble et al., 2017). MCT1 is a major transporter that regulates lactate efflux from ECs and maintains homeostasis in the intracellular environment (Végran et al., 2011). When impaired, its function aggravates lactate accumulation in ECs and reduces glycolysis flux. We found that THSWD could significantly enhance the glucose uptake and release of pyruvate and lactate in BMECs. Moreover, the ATP expression level was also significantly increased. Compared to the THSWD group, the 2DG + THSWD group suppressed glycolysis levels, suggesting that THSWD could effectively activate glycolysis in BMECs and increase its levels. As previously described, THSWD could effectively promote the mature angiogenesis process in BMECs, potentially related to its increased glycolysis flux. The results of WB assays unveiled that THSWD effectively increased the expression of GLUT1, glycolysis kinases (HXK1, PKM, PFKFB3), and MCT1. These findings suggest that THSWD may effectively increase glycolysis by up-regulating the content of critical proteins and enzymes in the glycolysis pathway and promoting the expression of mature angiogenic factors, accelerating the process of mature angiogenesis.

As described previously, THSWD for IS treatment is characterized by multiple pathways, multiple targets, and limited side effects. This can compensate for the issues of single targets and numerous side effects present in the Western medical treatment of IS. This study offers a new research strategy for treating IS in THSWD. However, it can be improved via the following aspects: 1) examining the mature angiogenesis at different time points (days 3, 14, and 21); 2) using EC glycolysis gene knockout mice for subsequent validation; 3) additional IS patient clinical data.

## Conclusion

These findings suggest that THSWD may exert a critical protective influence in CIRI by promoting mature angiogenesis via activation of the glycolysis pathway. Therefore, promoting mature angiogenesis is an effective therapeutic strategy for IS. In addition, this study offers a theoretical basis for preclinical studies of the TCM compound THSWD in treating IS.

## Data Availability

The original contributions presented in the study are included in the article/Supplementary Material, further inquiries can be directed to the corresponding authors.

## Abbreviations

IS, Ischemic stroke; ECs, Endothelial cells; TCM, Traditional Chinese medicine; THSWD, Taohong Siwu Decoction; CIRI, Cerebral ischemia-reperfusion injury; MCAO/R, Middle cerebral artery occlusion/reperfusion; OGD/R, Oxygen-glucose deprivation/reoxygenation; TTC, 2,3,5-triphenyltetrazolium chloride; HE, Hematoxylin-eosin; 2DG, 2-Deoxy-D-glucose; NBP, Butylphthalide; UPLC MS/MS, Ultra-performance chromatography-mass spectrometry; SD, Sprague-Dawley; ELISA, Enzyme-linked immunosorbent assay; mNSS, Modified neurologic severity score; WB, Western Blotting; BMECs, Brain microvascular endothelial cells.
