# Supplementary figures and images for: Promotion of mature angiogenesis in ischemic stroke by Taohong Siwu decoction through glycolysis activation
Source: Front Pharmacol. 2024 Jun 18;15:1395167. doi: 10.3389/fphar.2024.1395167 (PMC11221195; doi:10.3389/fphar.2024.1395167)

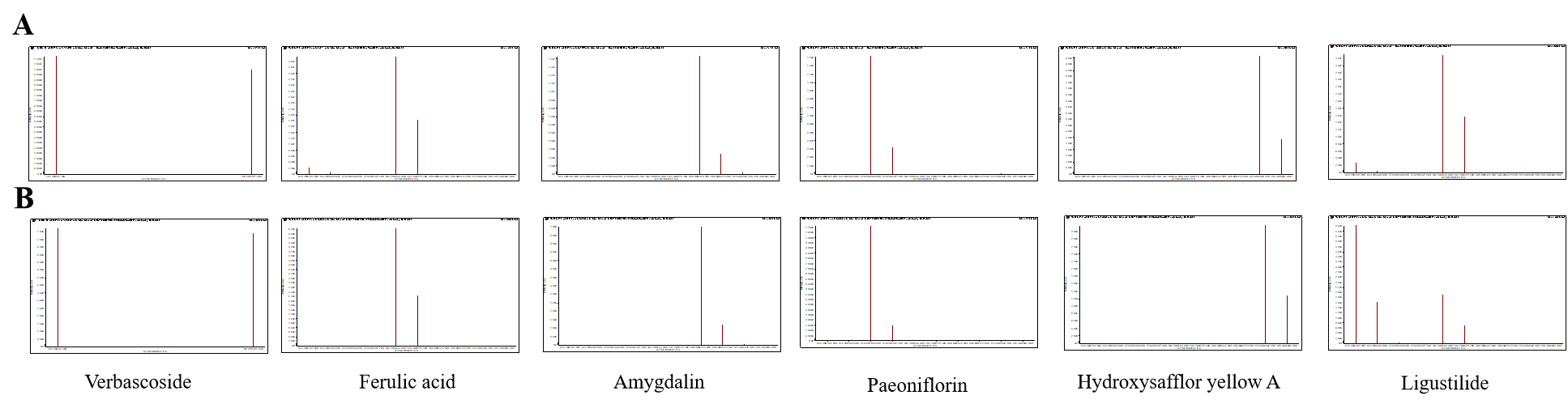

Supplement: Supplementary file 1 [file Image1.tif]
