# Supplementary material for: Promotion of mature angiogenesis in ischemic stroke by Taohong Siwu decoction through glycolysis activation
Source: Front Pharmacol. 2024 Jun 18;15:1395167. doi: 10.3389/fphar.2024.1395167 (PMC11221195; doi:10.3389/fphar.2024.1395167)
Supplement: Supplementary file 2 [file DataSheet1.pdf]

Supplemental material 1

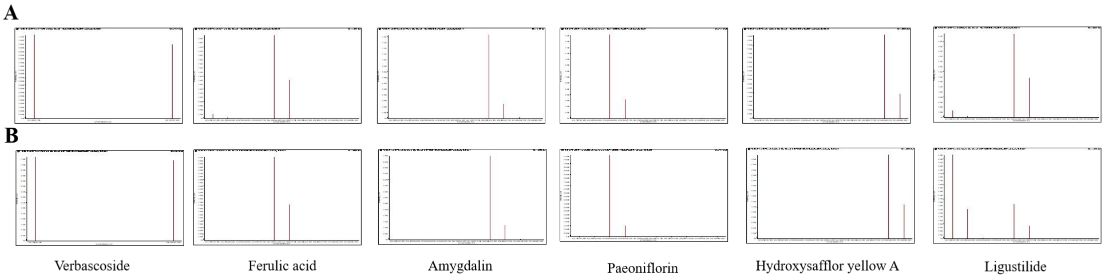

The Mass spectrum of six representative components of the mixed standard compounds (A) and THSWD (B).
