# Supplementary material for: Promotion of mature angiogenesis in ischemic stroke by Taohong Siwu decoction through glycolysis activation
Source: Front Pharmacol. 2024 Jun 18;15:1395167. doi: 10.3389/fphar.2024.1395167 (PMC11221195; doi:10.3389/fphar.2024.1395167)

Fig. 2C TTC staining

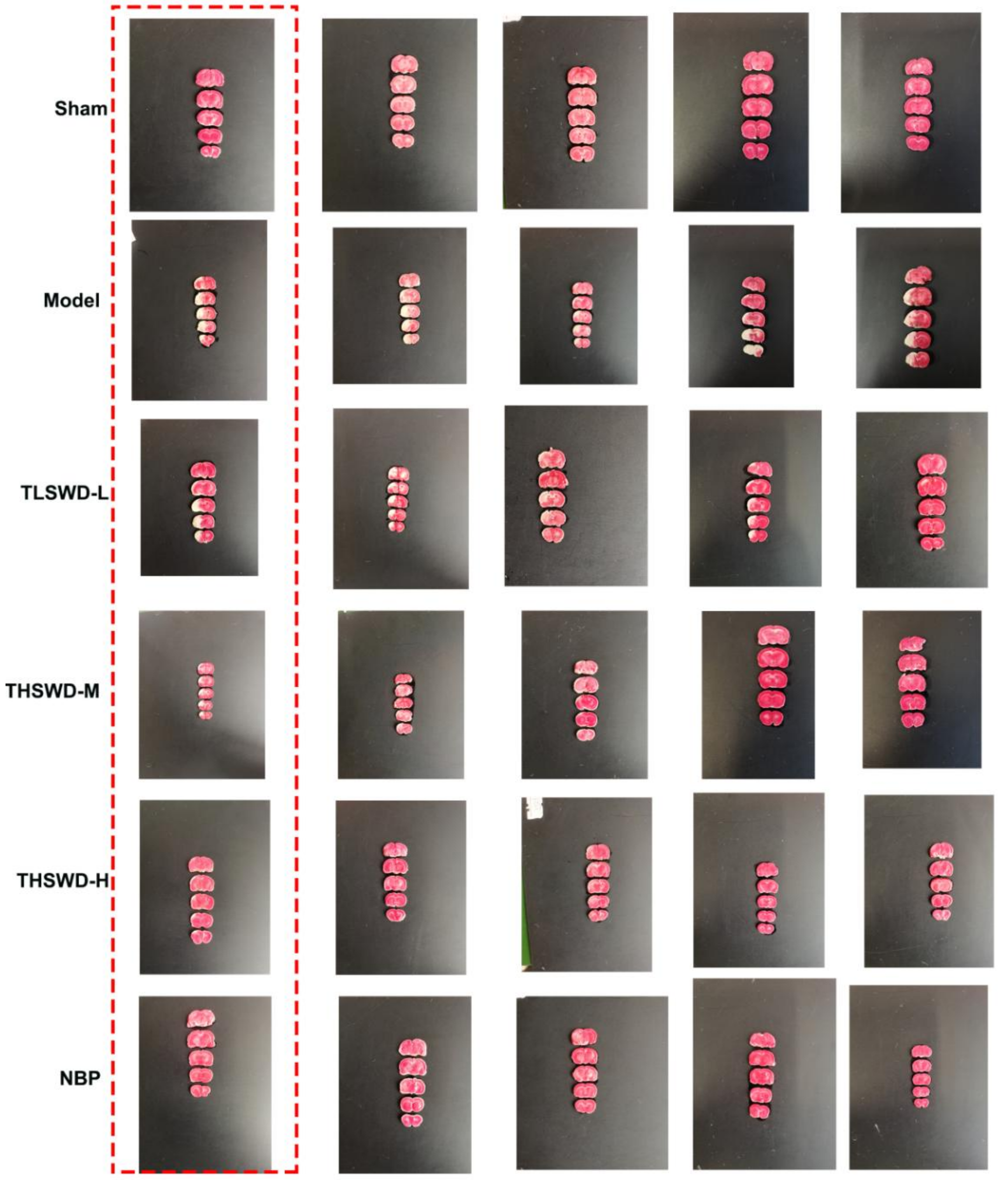

Fig. 2E HE staining

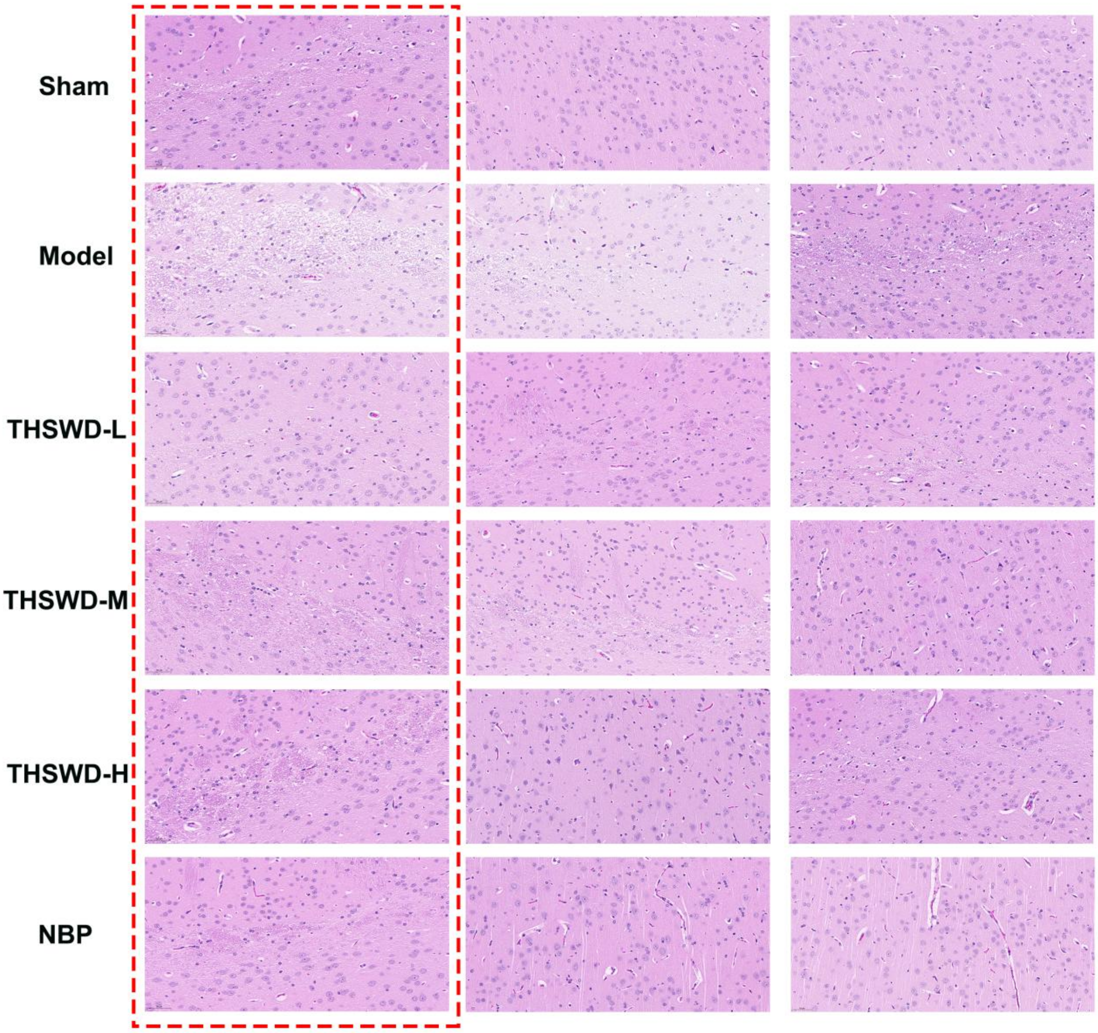

Fig. 2F Nissl staining

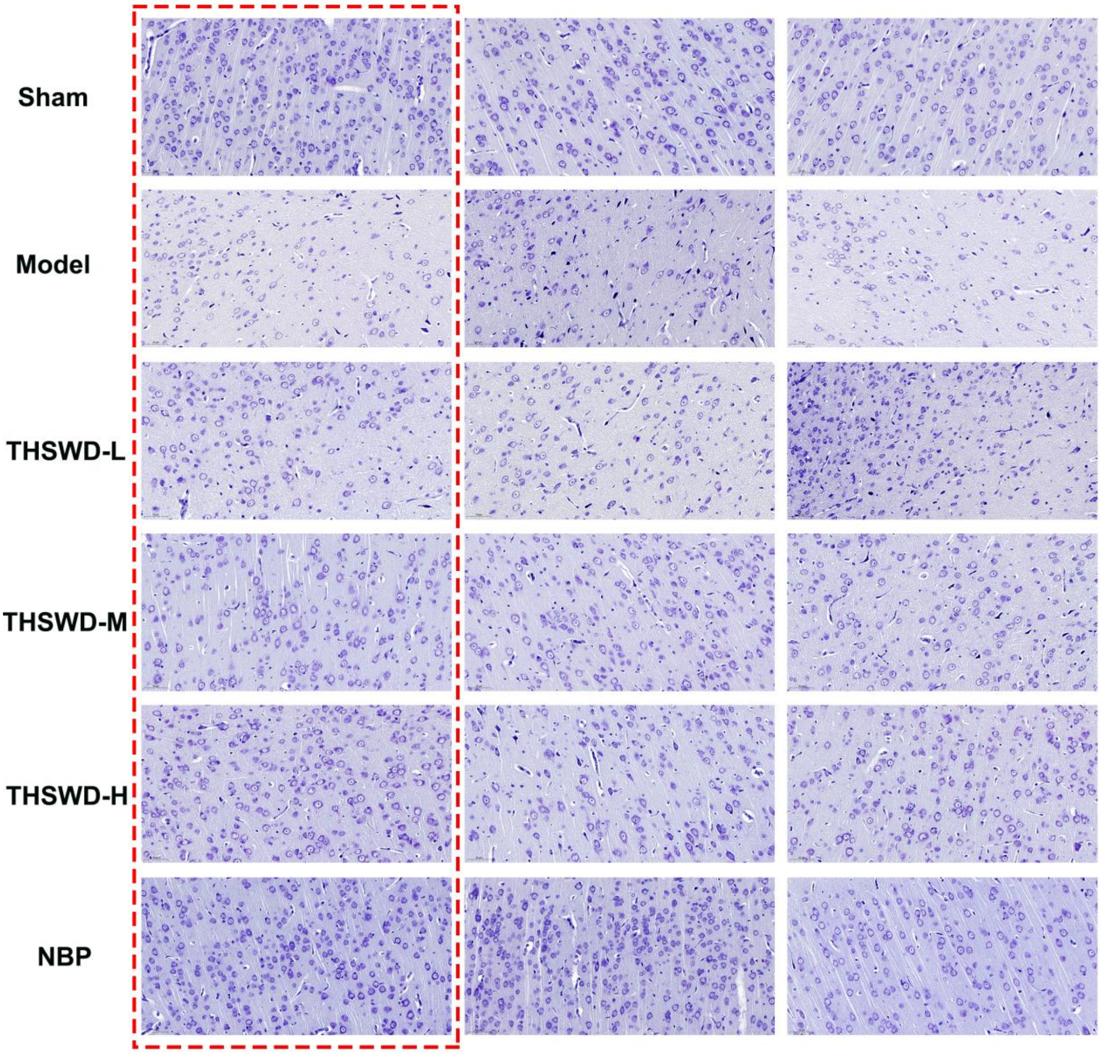

Fig.3A

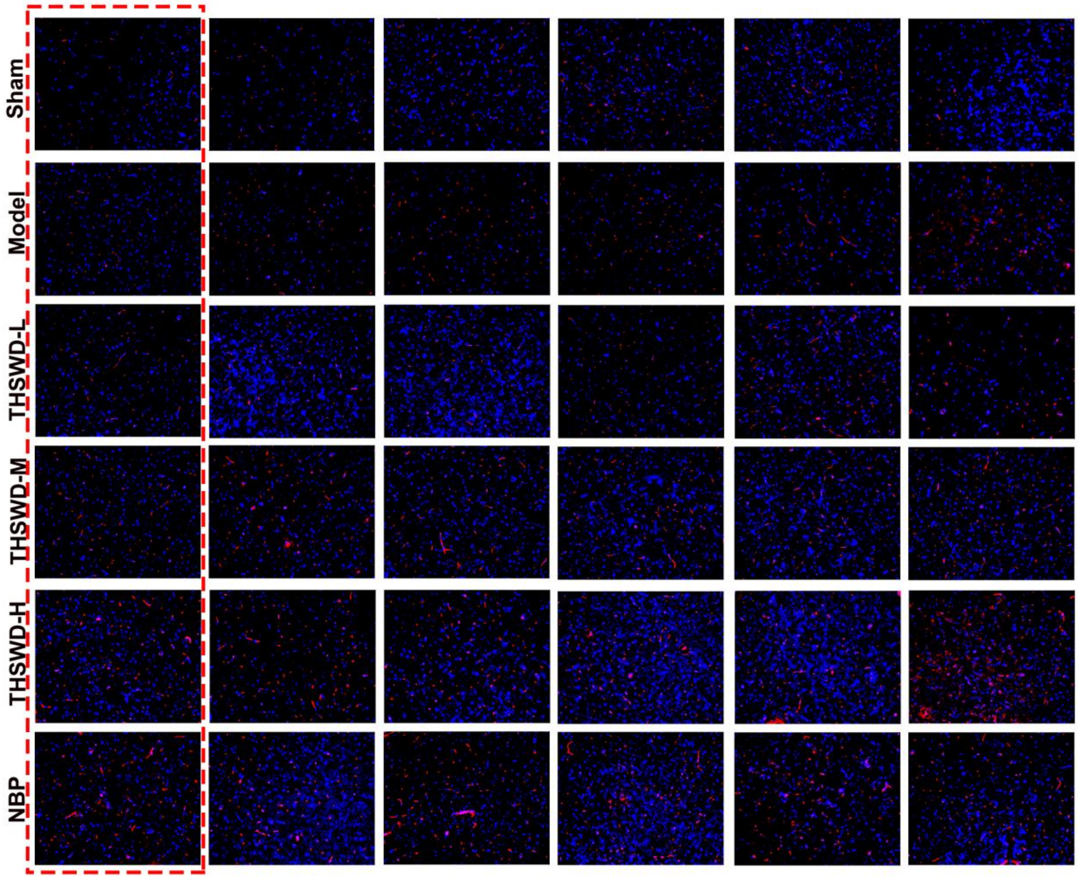

Fig. 4A Ang1

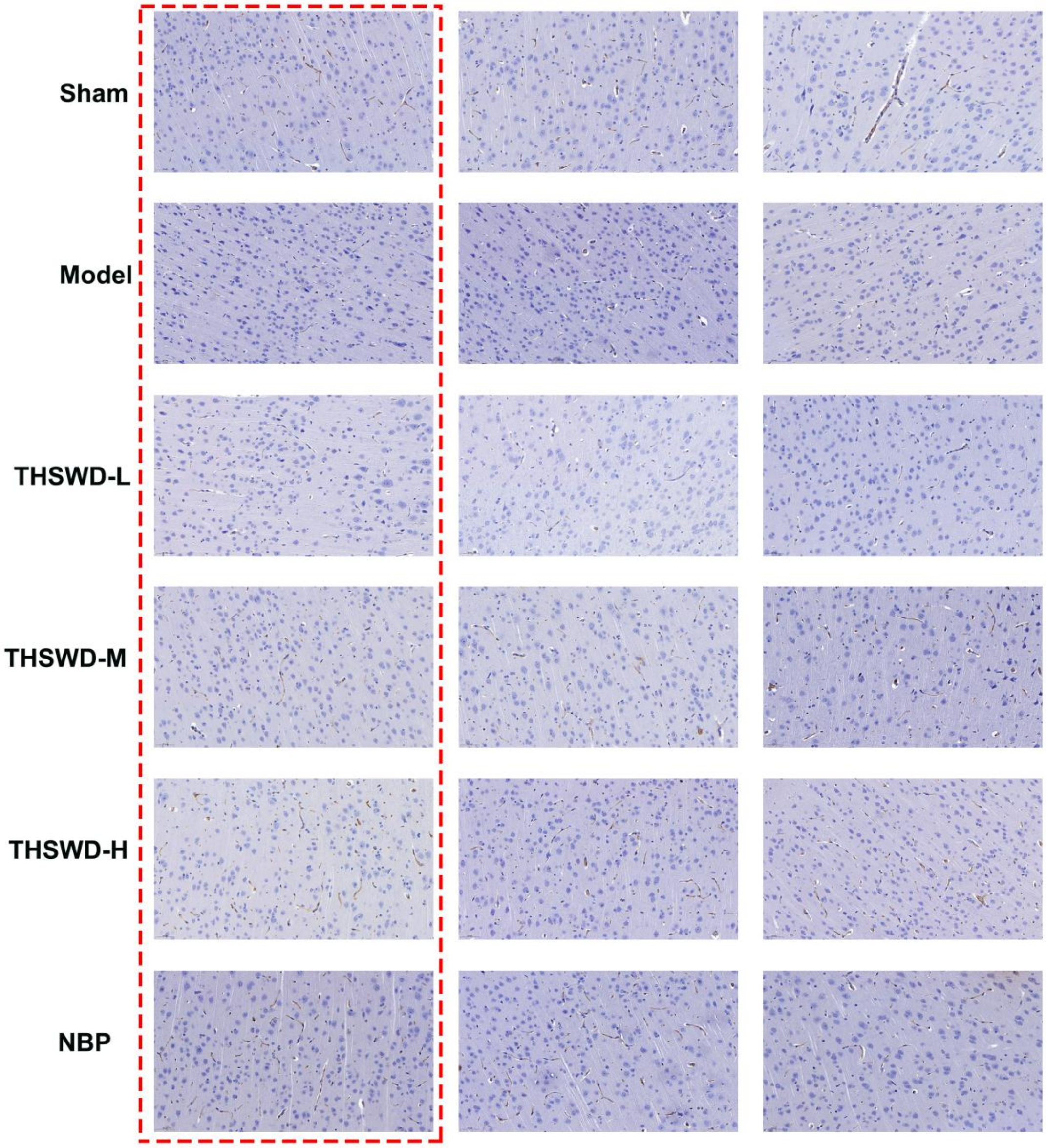

Fig. 4B PDGFB

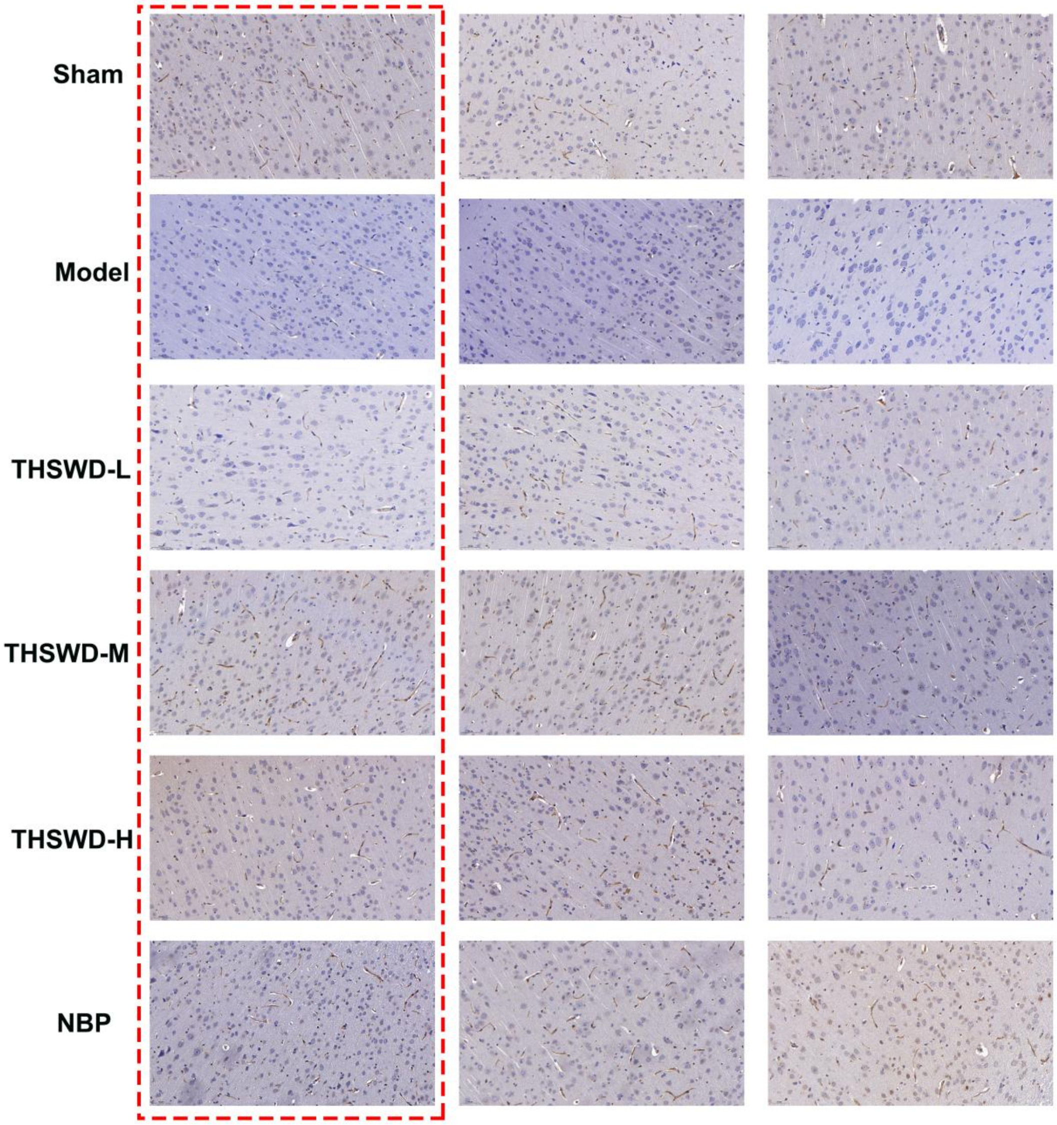

Fig. 4C PDGFRβ

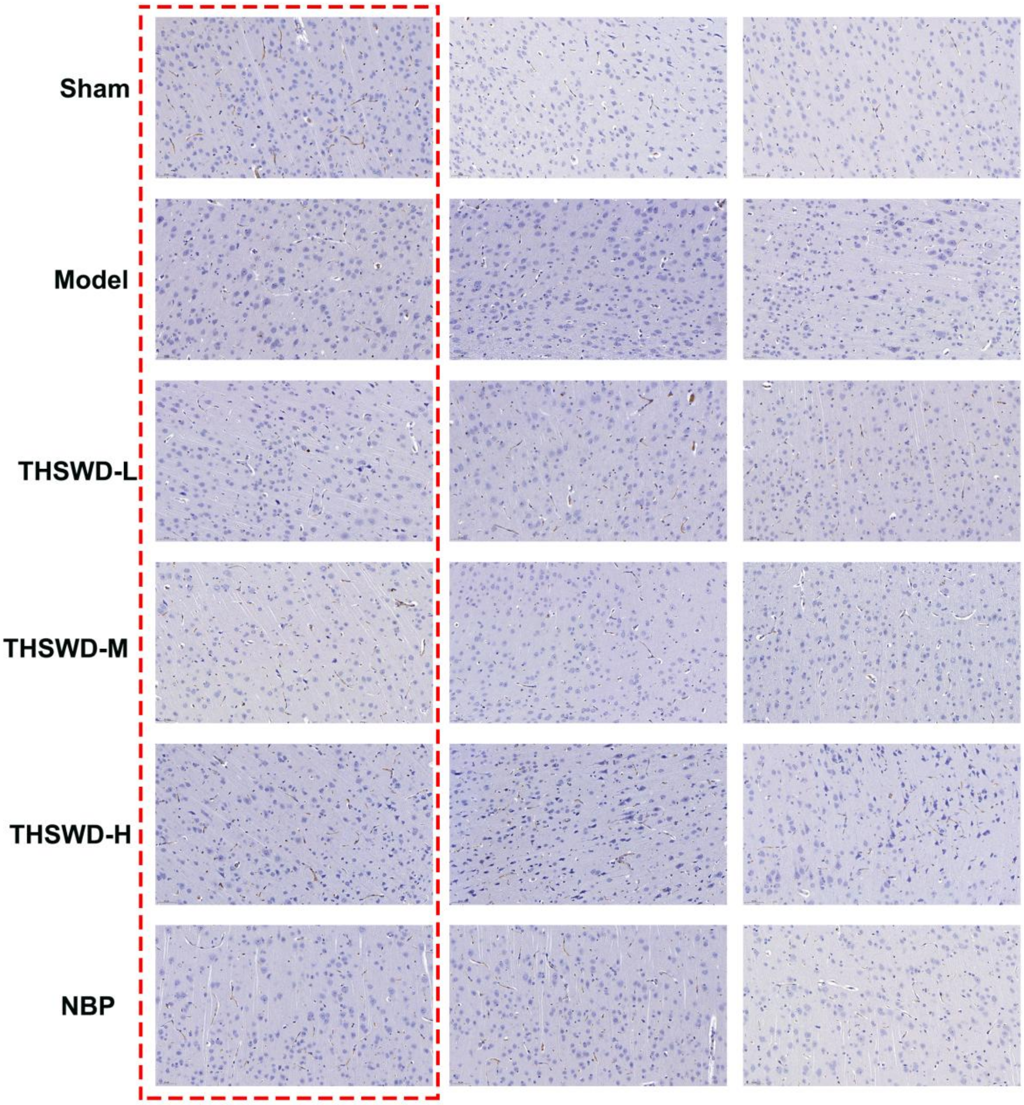

Fig. 7B Wound healing

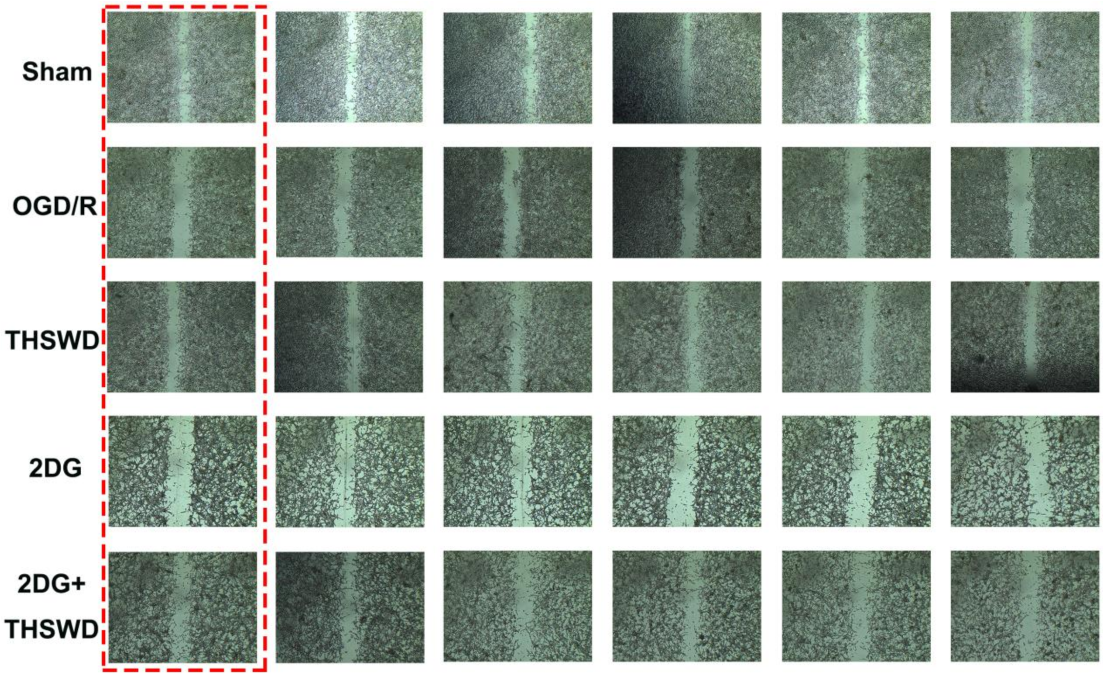

Fig. 7C Sprouting assay

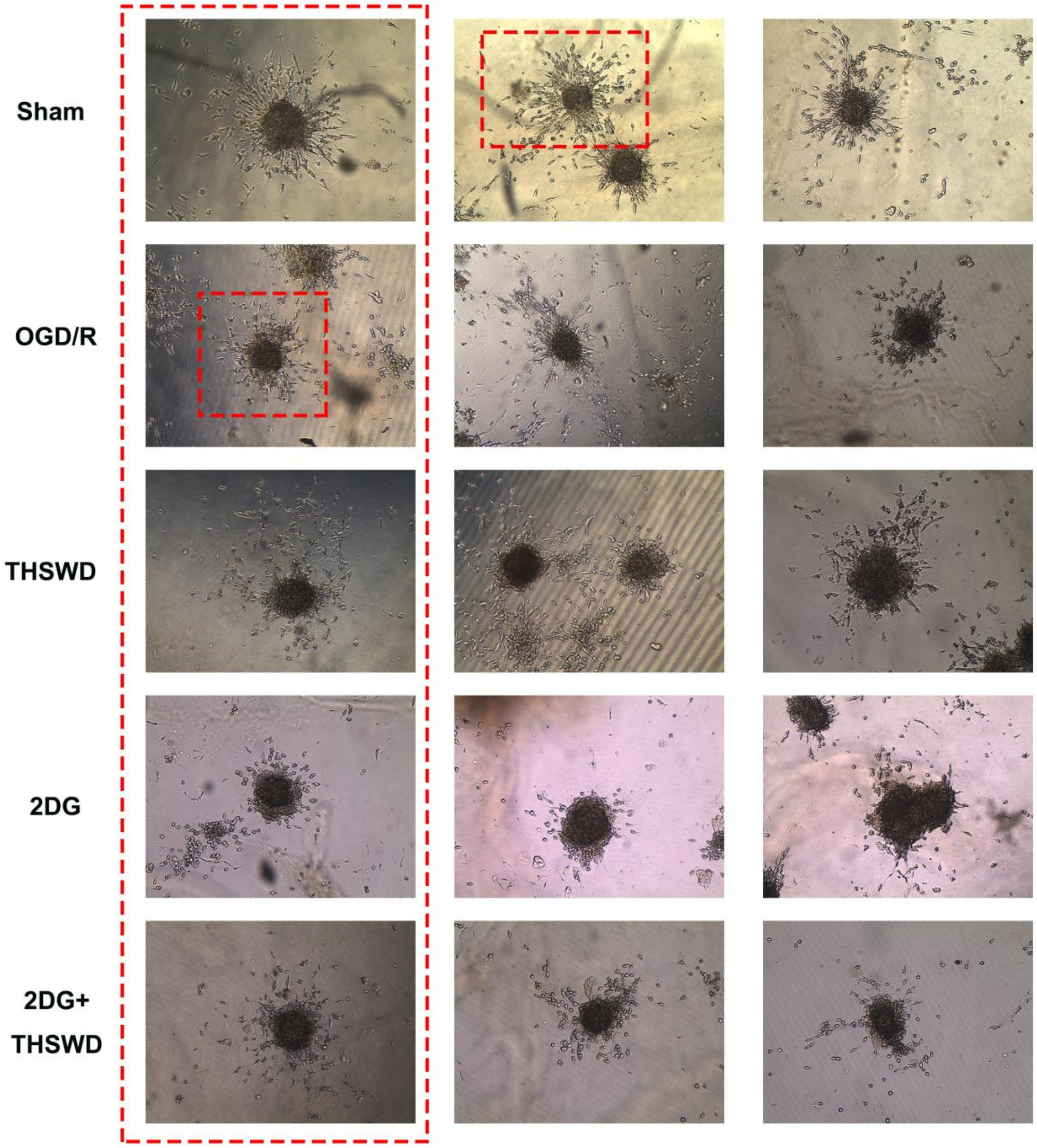

Fig. 7D Tube formation assay

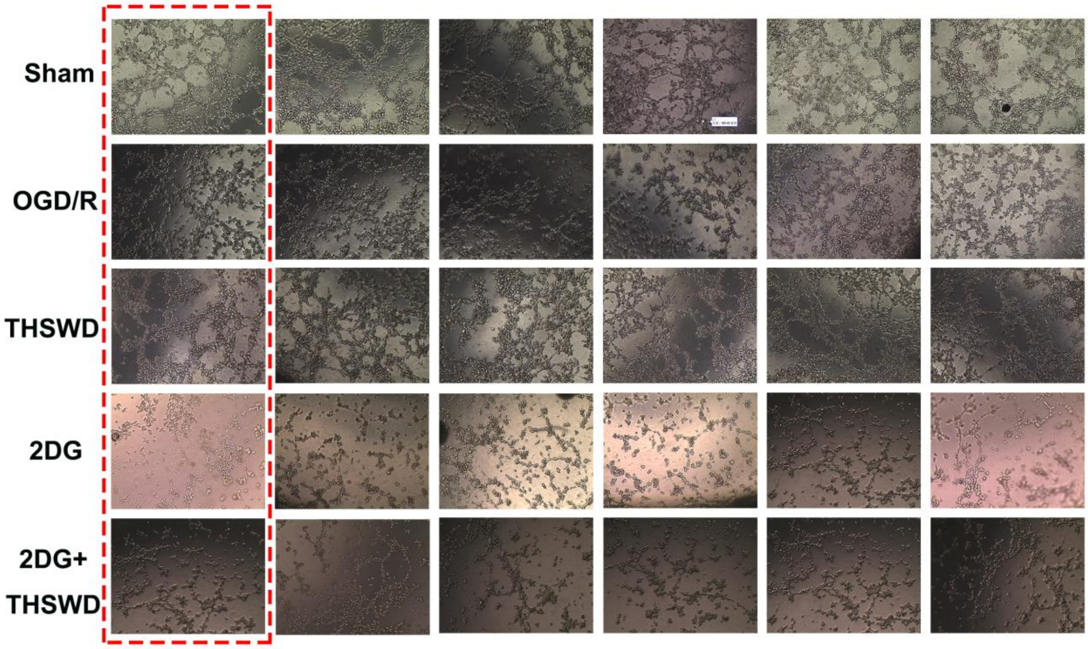

Fig. 7E Pericyte recruitment

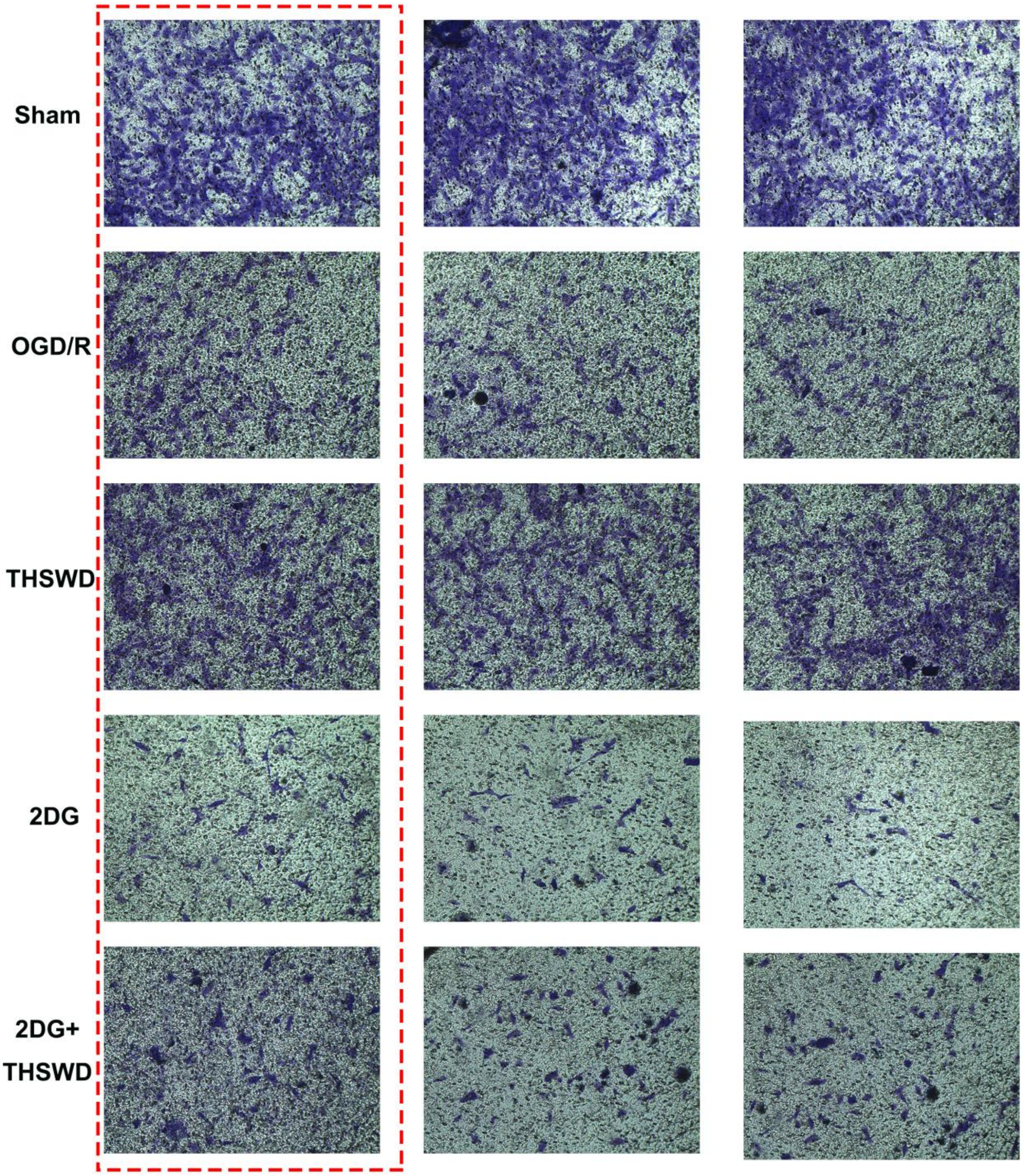

Fig. 8D Western blotting

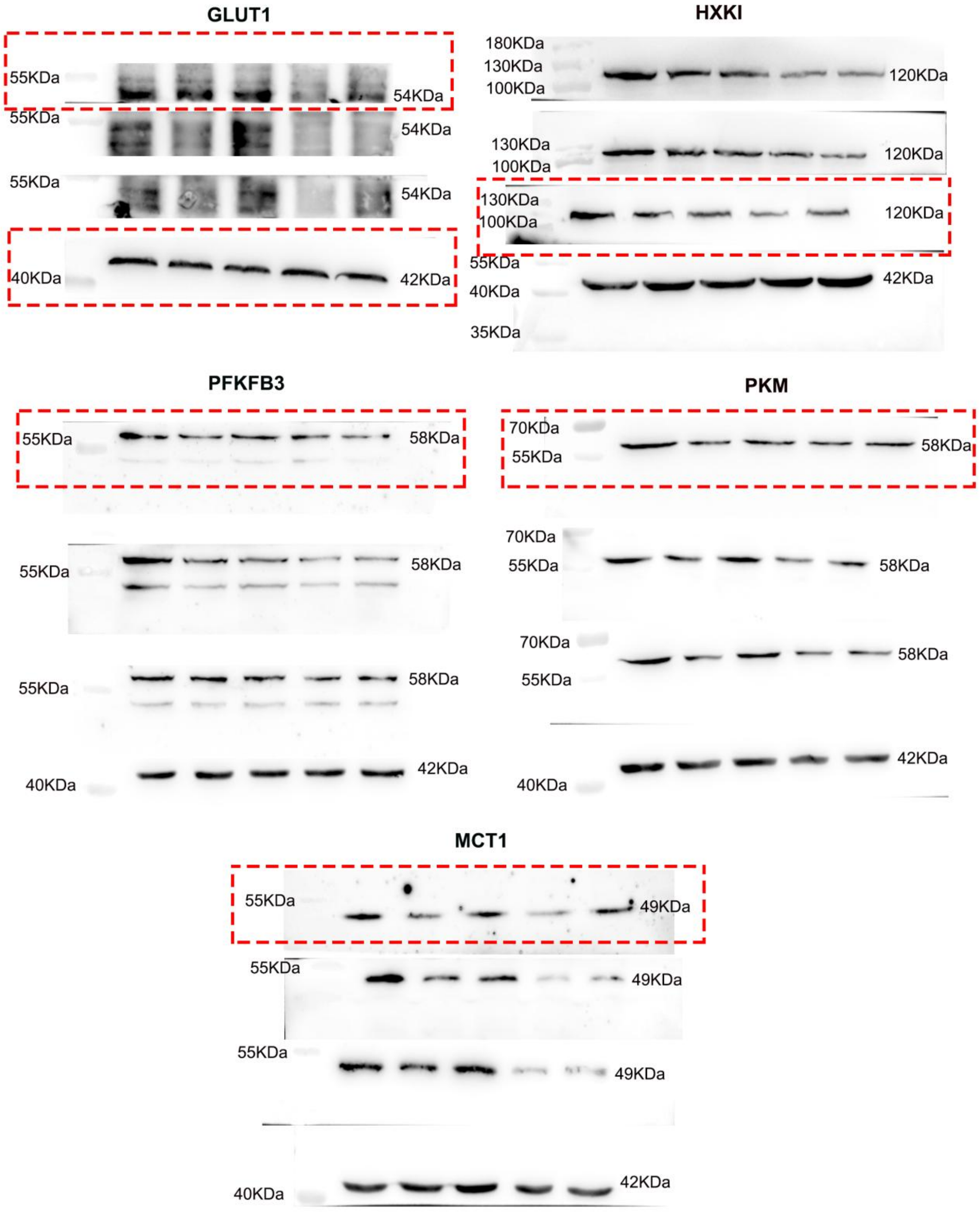

Supplement: Supplementary file 3 [file DataSheet2.zip › Original data-figure.pdf]
